# Hv1 inhibition rescues AD pathology by restoring microglial mitochondrial function and enhancing mitochondrial transfer

**DOI:** 10.1038/s12276-025-01593-z

**Published:** 2025-12-17

**Authors:** Jiayuan Lin, Huayun Han, Kexin Wu, Xingyu Wu, Juwen Shen, Yiqing Mo, Qiansen Zhang, Huaiyu Yang, Zhihua Yu

**Affiliations:** 1 Department of Pharmacology and Chemical Biology, Shanghai Jiao Tong University School of Medicine, Shanghai, China; 2 Shanghai Key Laboratory of Regulatory Biology, Institute of Biomedical Sciences and School of Life Sciences, East China Normal University, Shanghai, China; 3 Suzhou Institute of Drug Innovation, Shanghai Institute of Materia Medica, Chinese Academy of Sciences, Suzhou, China

**Keywords:** Alzheimer's disease, Neurodegenerative diseases

## Abstract

Hyperphosphorylated tau aggregation and neuroinflammation are hallmark pathologies of Alzheimer’s disease (AD), with microglia playing a critical role in modulating these processes through maintaining immune homeostasis and clearing pathological tau, both of which depend on mitochondrial health. However, the mechanisms underlying microglial mitochondrial dysfunction in AD remain poorly understood, limiting therapeutic development. Hydrogen voltage-gated channel 1 (Hv1), expressed in microglia within the central nervous system, regulates intracellular pH and reactive oxygen species generation. Here we observe that Hv1 is upregulated in activated microglia in AD mouse models. Remarkably, Hv1 contributes to electron transport chain abnormalities, leading to mitochondrial oxidative stress, loss of mitochondrial membrane potential, impaired ATP production and deficient mitophagy in tau pathology. These deficits impair tau clearance through phagocytosis and autophagy but can be significantly reversed by the Hv1-specific inhibitor YHV98-4. Furthermore, YHV98-4 enhances microglia-to-neuron mitochondrial transfer, promoting the delivery of functional mitochondria to rescue neuronal damage and improve cognitive function. Collectively, our study underscores the pivotal role of Hv1 in microglial mitochondrial dysfunction in AD and identifies YHV98-4 as a promising therapeutic candidate.

## Targeting Hv1 restores mitochondrial function in Alzheimer’s

Alzheimer’s disease (AD) is a major cause of dementia, affecting millions worldwide. Researchers are exploring new ways to understand and treat AD. A recent study focuses on microglia, the brain’s immune cells, and their role in AD. They found that a protein channel called Hv1 is more active in microglia during AD, leading to harmful inflammation and neuron damage. The study used mice with AD-like symptoms to test a new drug, YHV98-4, which blocks Hv1. Researchers injected this drug into the mice’s brains and observed its effects. They found that YHV98-4 reduced inflammation, improved mitochondrial function and helped microglia transfer healthy mitochondria to damaged neurons. This process protected neurons and improved cognitive function in the mice. The results suggest that targeting Hv1 with YHV98-4 could be a promising treatment for AD.

This summary was initially drafted using artificial intelligence, then revised and fact-checked by the author.

## Introduction

By 2050, an estimated 152.8 million people worldwide are projected to suffer from dementia^1^. Alzheimer’s disease (AD), which accounts for 60–70% of dementia cases globally, is associated with high incidence, significant mortality and a substantial socioeconomic burden^2^. As populations continue to age, the demand for effective treatments will inevitably rise. However, the absence of curative therapies underscores the urgent need to deepen our understanding of AD pathogenesis and develop novel intervention strategies.

Microglia, the primary innate immune cells responsible for maintaining homeostasis in the central nervous system (CNS), play a pivotal role in AD pathogenesis^2^. Under AD pathological conditions, microglia become activated, releasing reactive oxygen species (ROS) and proinflammatory factors while promoting T cell infiltration and activation^3^. This dysregulated immune microenvironment exacerbates neuroinflammation and accelerates neuronal loss. Moreover, activated microglia contribute to tau phosphorylation and propagation^4^. Mitochondria are essential for maintaining microglial function, not only by generating ATP to sustain cellular activity but also by regulating redox balance^5–7^. They also play a crucial role in immune homeostasis, as mitochondrial DNA leakage can trigger immune activation and drive inflammation^8,9^. Mitochondrial dysfunction, widely observed in AD, is closely associated with microglial activation and functional impairment^6,9–12^. Furthermore, recent studies suggest that healthy microglia can transfer mitochondria to rescue damaged neurons through tunneling nanotubes (TNTs). However, the extent of mitochondria transfer under AD pathology and its impact on neuronal health remain unclear^13^. Given these findings, targeting mitochondrial dysfunction presents a promising therapeutic strategy for AD.

The hydrogen voltage-gated channel 1 (Hv1) channel, encoded by the *Hvcn1* gene, is a voltage-gated proton channel that facilitates proton transport across membranes, driven by proton concentration gradients^14^. Highly expressed in immune system cells, Hv1 primarily regulates intracellular pH and ROS generation^15^. In the CNS, Hv1 expression was first identified in microglia in 2012, with no detectable expression in central neurons or astrocytes. It has been implicated in ROS generation and inflammation, exacerbating stroke pathology^16^. In addition, Hv1 has been linked to the progression of CNS diseases such as brain injury, demyelinating diseases and Parkinson’s disease^14,17,18^. Given the critical role of microglial oxidative stress in AD, investigating Hv1’s involvement in AD pathogenesis is of particular interest. Moreover, as mitochondria are the primary sources of ROS and depend on a stable proton gradient for proper function, the impact of Hv1 on the electron transport chain (ETC) and mitochondrial function in microglia warrants further exploration.

In this study, we find the upregulation of Hv1 in activated microglia in AD mouse models, where it contributes to tau hyperphosphorylation, microglia-mediated T cell infiltration and neuroinflammation. Notably, we indicate the critical role of Hv1 in mitochondrial damage in AD. Inhibiting Hv1 with the selective inhibitor YHV98-4 mitigates ETC deficiency in tauopathy, especially in complexes I and V, thereby reducing oxidative stress, rescuing mitophagy deficiency and restoring mitochondrial function. Furthermore, while the mitochondrial transfer is impaired in tauopathy, YHV98-4 notably enhances microglia-to-neuron mitochondrial transfer, facilitating the donation of functional mitochondria. As a result, YHV98-4 effectively alleviates AD pathology, protects neurons and improves cognitive function. Together, our study reveals Hv1’s role in microglial mitochondrial dysfunction in AD and proposes YHV98-4 as a promising therapeutic candidate.

## Methods

### Animals

*Hvcn1*^–/–^ mice were generated as previously described^19^. C57BL/6 mice were obtained from Shanghai SLAC Laboratory. Seven-month-old 3×Tg mice were obtained from Jackson Laboratory (#34830-JAX). Both male and female mice were used in the experiments. All mouse experiments were conducted based on the relevant guidelines and approved by the Animal Experimentation Ethics Committee of Shanghai Jiao Tong University School of Medicine (Shanghai, China). Mice were housed at 23 °C ± 2 °C with a 12 h light–dark cycle and provided with food and water ad libitum.

### Preparation of PHF

Paired helical filament (PHF) was prepared as previously described^20^. In brief, recombinant human microtube-associated protein tau monomers (C521979, Sangon Biotech) were dissolved in phosphate-buffered saline (PBS) (LS2041-500, Bioagrio) at a concentration of 50 μM, then incubated with 2 mM dithiothreitol (B645939, Sangon Biotech), protease inhibitor cocktail (P1025, Beyotime) and 12.5 μM heparin lithium salt (A690013, Sangon Biotech) to induce fibril assembly for 2 weeks at 37 °C. To maintain reducing conditions in the reaction system, 1 mM dithiothreitol was added every 24 h. After fibril assembly, the remaining free heparin and dithiothreitol were removed by dialysis with PBS for 24 h. PHF was validated by transmission electron microscopy and stored at −80 °C.

### Cell cultures

BV2 cell line was purchased from the Chinese Academy of Sciences Cell Bank. The cells were grown in Dulbecco’s modified Eagle’s medium (DMEM) (L110KJ, BasalMedia) supplemented with 10% fetal bovine serum (S711-001S, Shuangru Biotechnology), 1% penicillin–streptomycin (C0222, Beyotime) and 1% glutamax (35050061, Gibco) at 37 °C with 5% CO_2_.

### Preparation of the diseased-associated microglia

PHF was utilized to model tauopathy, as previously described^21^. To obtain more toxic tau species, PHF was depolymerized by ultrasound into tau oligomers. BV2 cells were then incubated with the post-ultrasonic PHF to mimic diseased-associated microglia.

### YHV98-4 treatment

YHV98-4 was synthesized and characterized as previously described^19^. For in vivo treatment, YHV98-4 was dissolved in a solution consisting of 10% dimethyl sulfoxide (196055, MP Biomedicals), 10% Tween-80 (A600562, Sangon Biotech) and 80% (20% sulfobutylether-β-cyclodextrin) (S11013, Yuanye Bio-Technology). YHV98-4 and vehicle controls were administered via intraperitoneal injections at a fixed time every day, continuing treatment for 21 days for intrahippocampal injection of AAV-hTAU mice and 30 days for 3×Tg mice (10 mg/kg intraperitoneal injection). For in vitro treatments, YHV98-4 was dissolved in dimethyl sulfoxide at a concentration of 40 mM and then diluted in DMEM to a concentration of 20 μM before use

### Primary neuronal cultures

Primary neuronal cultures were conducted as previously described^22^. In brief, brains from newborn C57BL/6 mice (P0) were taken out, rinsed in DMEM for three times and then stripped of the meninges. Hippocampus was dissected and cut into small pieces, then digested in papain and DNase I (DN25, Sigma-Aldrich) at 37 °C with 5% CO_2_ for 15 min. A total of 1.5 × 10^5^ neurons then were seeded in PLL-coated four-well dishes per well and grew in Neurobasal-A medium (10888022, Gibco) supplemented with 2% B-27 (17504022, Gibco), 1% penicillin–streptomycin (C0222, Beyotime) and 1% glutamax (35050061, Gibco) at 37 °C with 5% CO_2_.

### Primary microglia cultures

Primary microglia cultures were conducted as previously described^22^. In brief, mixed glial cultures were obtained from newborn C57BL/6 mice (P2). Brain tissues were digested with trypsin and DNase I at 37 °C for 10 min. Cells were resuspended in DMEM (L110KJ, BasalMedia) supplemented with 10% fetal bovine serum (S711-001S, Shuangru Biotechnology), 1% penicillin–streptomycin (C0222, Beyotime) and 1% glutamax (35050061, Gibco) and seeded in PLL-coated T75 flask. On day 10, primary microglia were obtained from the mixed glial cultures by shaking at 200 rpm for 4 h, then plated according to the experiment. Microglia-conditioned medium treated with 1 μg/ml PHF with or without 20 μM YHV98-4 was collected.

### Viral vectors

AAV2/8-CAG-MCS-EGFP-3FLAG (AAV–EGFP) (titer: 4.09 × 10^13^ vector genome (v.g.)/ml, dilution: 1:4, Obio Technology) and AAV2/8-CAG-TAU-EGFP-3FLAG-WPRE (AAV-hTAU) (titer: 3.14 × 10^12 ^v.g./ml, Obio Technology) were aliquoted and stored at −80 °C.

### Stereotaxic injection

Stereotaxic injection was performed as previously described^23^, with a few modifications. In brief, 2-month-old C57BL/6 mice as well as *Hvcn1*^–/–^ and wild-type (WT) littermates were anesthetized before receiving a hippocampal injection of 1.5 μl of AAV–EGFP or AAV-hTAU at the following coordinates: anterior–posterior（AP: −2.0 mm, medial–lateral（ML: ±1.50 mm and dorsal–ventral（DV: −2.0 mm relative to the bregma at a rate of 0.20 μl/min using a stereotaxic injection apparatus (RWD Life Science) and a needle syringe (Hamilton).

### Behavioral tests

Mice were gently petted daily for 2–3 min for 2 days before the experiment to alleviate fear responses. Moreover, they were familiarized with the testing room 1 day before the experiment to acclimate to the environment. Three-month-old C57BL/6, *Hvcn1*^–/–^ and littermate mice that received intrahippocampal injections of viral vectors were used for behavioral tests. Eight-month-old 3×Tg mice were subjected to behavioral tests. Age-matched C57BL/6 mice were employed as the control group.

### NOR test

A novel object recognition (NOR) test was conducted on two consecutive days in an open field box (40 cm × 40 cm × 35 cm) as previously reported^24^. In brief, on the first day, mice were placed in the center of the empty open field box and allowed to explore for 5 min for habituation. On the second day, mice were given 5 min to explore the box, which now contained two identical objects positioned equidistantly. After a 2 h interval, one of the objects was replaced by a different color and shape object. Mice were allowed to explore the open field box for another 5 min. The box was cleaned with 75% ethanol after each session. Mouse activities were automatically recorded and analyzed by EthoVision XT Version 16. The discrimination index was calculated as the time spent with the novel object divided by the sum of the time spent with the novel object and time spent with the familiar object, multiplied by 100%.

### Y-maze test

The Y-maze test was conducted using an apparatus consisting of three arms at 120° (40 cm × 10 cm × 15 cm) as previously described^25^. In brief, mice were placed in the center of the apparatus and allowed to explore the three arms for 5 min. Spontaneous alternations, defined as any three consecutive choices of three different arms without revisiting a previously explored arm, were automatically recorded and analyzed using EthoVision XT Version 16 software. The maze was cleaned with 75% ethanol between different tests to prevent odor interference.

### MWM test

The Morris water maze (MWM) test was conducted according to reported procedures^26^. In brief, the MWM apparatus consisted of a circular tank (120 cm × 50 cm) filled with opaque water maintained at a temperature of 20–22 °C. The hidden platform was positioned 1 cm below the water surface in a fixed quadrant. Distal cues in the form of four different shapes were attached to the walls. During the training trial, mice underwent four training trials from four different locations per day for five consecutive days, with varied placement orders. Mice were tasked with locating the hidden platform within 60 s. Those failing to find the platform within the allotted time were guided to it and allowed to stay for 10 s. On the probe test day (the sixth day), the hidden platform was removed, and mice underwent two training trials from two different starting locations. In each trial, mice were given 60 s to swim freely. EthoVision XT Version 16 was used to record and calculate various parameters including latency in each training trial, swimming speed, cumulative distance, trajectory, number of platform crossings and time spent in the target quadrant.

### Brain tissue preparation

At the final point, mice were anesthetized and perfused with precooled PBS followed by 4% paraformaldehyde. Brains were then removed and post-fixed in 4% paraformaldehyde at 4 °C for 1 day, cryoprotected in 30% sucrose for 2 days and subsequently embedded in optimal cutting temperature compound. The hippocampus was sectioned into 20-μm-thick coronal sections using a cryostat (Leica, CM1950) and stored at −20 °C.

### Immunofluorescence

For human brain sections embedded in paraffin, deparaffinization was achieved using xylene and ethyl alcohol, followed by antigen retrieval using sodium citrate. Frozen sections of mice hippocampus were blocked with 10% goat serum (E510009, Sangon Biotech) and 0.3% Triton X-100 (A110694, Sangon Biotech) in PBS for 90 min at room temperature. Subsequently, the sections were incubated with primary antibodies diluted in PBS containing 5% goat serum and 0.3% Triton X-100 overnight at 4 °C. The primary antibodies used were: anti-Iba1 (1:100, ab283346, Abcam), anti-Iba1 (1:200, A19776, ABclonal), anti-NeuN (1:500, ab279296, Abcam), anti-NeuN (1:400, ab104224, Abcam), anti-MAP2 (1:200, 8707, Cell Signaling Technology), anti-AT8 (phospho-Tau (S202/T205), 1:600, GB113883, Servicebio), anti-CD44 (1:500, GB112054, Servicebio), anti-Hv1(1:100, AHC-001, Alomone Labs), anti-CD11c (1:100, ab254183, Abcam), anti-mouse I-A/I-E (1:100, 107601, BioLegend), anti-nicotinamide adenine dinucleotide phosphate oxidase 2 (NOX2) (1:200, GB11391-100, Servicebio), anti-GFAP (1:500, BD12096, Servicebio), anti-CD4 (1:100, 100505, BioLegend) and anti-CD8 (1:200, GB114196, Servicebio) antibodies. After primary antibody incubation, sections were washed three times in PBS at room temperature for 5 min each. The sections were then incubated with secondary antibodies diluted in PBS containing 5% goat serum and 0.3% Triton X-100 for 1 h. The secondary antibodies used were: Alexa Fluor 647 anti-rabbit (1:1,000, A32728, Invitrogen), Alexa Fluor 647 anti-rat (1:1,000, ab150159, Abcam), Cy3 anti-rabbit (1:200, GB21303, Servicebio) and Cy3 anti-mouse (1:200, GB21301, Servicebio). After three washes in PBS, the sections were stained with 4,6-diamidino-2-phenylindole (DAPI) and mounted onto glass coverslips. Imaging was performed using laser scanning confocal microscope (LSCM) (Leica, TCS SP8) and (Nikon, AX R) and a fluorescence microscope (Leica, DM6B). Images quantification was performed using Fiji software (NIH).

To measure the colocalization of Hv1 with mitochondria, BV2 cells were cultured in a four-well dish and stimulated with 1 μg/ml PHF for 24 h, with or without co-incubation with YHV98-4. MitoTracker Red CMXRos (40741ES50, Yeasen) was diluted in 1× Hanks’ Balanced Salt Solution (HBSS) (LS2035-500, Bioagrio) to a concentration of 50 nM and then incubated with the cells for 30 min at 37 °C in the dark. Subsequently, the cells were co-incubated with Hoechst (C1011, Beyotime) followed by fixation with 4% paraformaldehyde for 15 min. After blocking, the cells were incubated with anti-Hv1 antibody (1:100, AHC-001, Alomone Labs) overnight at 4 °C, followed by incubation with Cy3 anti-rabbit secondary antibody (1:200, GB21303, Servicebio) for 1 h at room temperature. Finally, the cells were stained with DAPI for 10 min and imaged using LSCM (Leica, TCS SP8). To measure mitophagy, cells were stained with anti-TOM20 antibody (1:1,000, AF1717, Beyotime) and anti-LAMP2 antibody (1:400, ab25631, Abcam) overnight at 4 °C, followed by incubation with Cy3 anti-rabbit secondary antibody (1:200, GB21303, Servicebio) and Alexa Fluor 647 anti-mouse secondary antibody (1:500, A32728, Invitrogen). Colocalization was quantified using Fiji software.

For in vitro coculture experiments, the primary antibody anti-MAP2 (1:400, 4542, Cell Signaling Technology) was used to label neuronal dendrites, anti-Iba1 (1:100, ab283346, Abcam) was used to label microglia, and anti-TOM20 antibody (1:1,000, AF1717, Beyotime) and anti-NeuN (1:500, ab279296, Abcam) were used to measure the neuronal mitochondria, followed by incubation with Cy3 anti-rabbit secondary antibody (1:200, GB21303, Servicebio), Alexa Fluor 488 anti-rat secondary antibody (1:500, A11006, Invitrogen) and Alexa Fluor 647 anti-mouse secondary antibody (1:500, A32728, Invitrogen). Images were captured using LSCM (Nikon, AX R) and quantified using Fiji software.

### Three-dimensional reconstruction of microglia

To measure microglia morphology, Imaris software (BitPlane Scientific Software, Zurich, Switzerland) was utilized for the analysis and three-dimensional (3D) reconstruction of Iba1^+^ microglia. The filament function was employed for individual cell analysis, and microglia were automatically reconstructed based on the Iba1 signal. Parameters such as microglial area, volume, filament length and the number of segment branches and terminals were recorded for analysis.

### Western blotting

Western blotting was conducted as previously described^27^. Lysates from different mice hippocampal tissues were obtained with 400 μl RIPA lysis buffer (P0013B, Beyotime) containing 1% phenylmethylsulfonyl fluoride protease inhibitor (ST506, Beyotime). The BCA Protein Concentration Assay Kit (P0010S, Beyotime) was used to guarantee an equal amount of protein lysates. The proteins were then transferred to polyvinylidene difluoride membranes and blocked with 5% skim milk for 1 h at room temperature, followed by incubation with primary antibodies: anti-β-actin (1:1000, AA128-1, Beyotime), anti-AT8 (1:700, GB11383, Servicebio) and anti-LC3B (1:1000, 2775, Cell Signaling Technology). Membranes were then incubated with the horseradish peroxidase secondary antibody for 1 h. Proteins were visualized using an enhanced ECL chemiluminescence substrate kit (36222ES76, Yeasen) and Image Studio Lite Version 5.2 software (LI-COR Biosciences).

### Measurement of ATP

The ATP level was measured using the enhanced ATP Assay Kit (S0027, Beyotime) following the manufacturer’s instructions. In brief, for the in vivo study, the lysis buffer was added at a ratio of 5 μl lysis buffer per milligram hippocampus. The sample was then homogenized and centrifuged, and the supernatant was collected for subsequent detection. For the in vitro study, BV2 cells were treated with 1 μg/ml PHF with or without 20 μM YHV98-4 for 24 h. Subsequently, the culture medium was replaced by lysis buffer at a volume equivalent to 1/10 of the culture medium. After centrifugation, the supernatant was collected. A total of 100 μl ATP detecting buffer was added to each well, incubated for 5 min and mixed with 20 μl of sample or the standard solution. Luminescence was detected using a microplate Reader (Thermo Scientific). The ATP level was normalized to the protein concentration, and the ATP level of the control group was further normalized to 1.

### Hematoxylin and eosin staining

Fresh tissues were fixed in 4% paraformaldehyde for 24 h. Subsequently, the tissues underwent dehydration, wax leaching and embedding before being cut into serial sections at 4 μm thickness. After dewaxing and hydration, the sections were stained with hematoxylin and eosin solution and observed under an optical microscope (NIKON, ECLIPSE E100, Japan).

### Flow cytometry

Mice were anesthetized followed by cardiac perfusion with 20 ml of precooled PBS to ensure that the isolated immune cells were from the brain parenchyma. The cortex and hippocampus were dissected and mechanically dissociated over a 70 μm cell strainer. After centrifugation, the supernatant was carefully removed. Subsequently, the remaining pellet underwent digestion with collagenase IV (40510ES60, Yeasen) and DNase I (DN25, Sigma-Aldrich) for 20 min (ref. ^28^). The digestion was terminated by adding equal volumes of DMEM with 10% fetal bovine serum. After centrifugation, the pellet was resuspended in 4 ml of a 37% stock isotonic Percoll (SIP) solution (CP8331, Coolaber), prepared by mixing Percoll with 10× HBSS (H1046, Solarbio) at a ratio of 9:1. This suspension was then diluted with 1× HBSS (LS2034-500, Bioagrio) to prepare the Percoll gradients. Subsequently, the resuspended pellet was carefully transferred to the top of 4 ml of 70% SIP, followed by laying 4 ml of 30% SIP on the top of the 37% SIP layer without intermixing. The sample was centrifuged with the lowest acceleration setting and no brake. The white floating myelin debris at the top layer was discarded, and the cell interphase between 37% SIP layer and 70% SIP layer was collected^29^. To exclude dead cells, the cell pellet was incubated with the Zombie Aqua Fixable Viability Kit (1:200, 423101, BioLegend) and then stained with antibodies including APC/Fire 750 anti-mouse CD45 (1:500, 103154, BioLegend), APC anti-mouse/human CD11b (1:500, 101211, BioLegend), PerCP/Cyanine5.5 anti-mouse TCR β chain (1:500, 109228, BioLegend), Pacific Blue anti-mouse CD4 (1:500, 100534, BioLegend), PE/Cyanine7 anti-mouse CD8a (1:500, 100722, BioLegend) and PE anti-mouse I-A/I-E (1:1,250, 107608, BioLegend). After washing with PBS containing 2% fetal bovine serum two times, the cells were resuspended and sorted using an LSRFortessa X-20 cell analyzer (BD Bioscience). A data analysis was conducted on FlowJo software (Tree Star).

### Magnet-activated cell sorting isolation of microglia

Magnet-activated cell sorting isolation of microglia was performed as previously reported^30^ with a few changes. CD11b (13-0112-82, Thermo Scientific) was incubated with Dynabeads (11047, Thermo Scientific) for 50 min to generate CD11b-conjugated magnetic beads, which were then placed on a magnetic column for 2 min, to remove the supernatant. The mice were killed, and the brains were immediately dissociated, with the cerebellum, midbrain and olfactory bulb removed. The brain tissue was then cut into fine pieces and digested by DNase I (DN25, Sigma-Aldrich) and papain at 37 °C with 5% CO_2_ for 17 min. After centrifugation at 800 rpm at 4 °C for 10 min, the supernatant was carefully removed, leaving 4 ml, which was subsequently used to resuspend the pellet. The cell suspension was added to CD11b-conjugated magnetic beads and incubated at 4 °C for 40 min for conjugation. The positive isolation of microglia was performed using a magnetic column and then purified by washing with buffer 2 (PBS with 1 mg/ml BSA and 0.5 mM EDTA) three times.

### RNA-seq

Total RNA from mice brains (excluding the cerebellum, midbrain, and olfactory bulb) was extracted using Trizol Reagent (15596018, Invitrogen), followed by quality control using a NanoDrop spectrophotometer (Thermo Scientific). For RNA sequencing (RNA-seq) library preparation, the NEBNext Ultra II RNA Library Prep Kit for Illumina was used for bulk RNA-seq, whereas the SMARTer Ultra Low Input RNA Kit was used for microtranscriptome sequencing. mRNA was purified using poly(T) oligo-attached magnetic beads and then cleaved using divalent cations in an Illumina proprietary fragmentation buffer. First-strand complementary DNA was synthesized using random oligonucleotides and SuperScript II, followed by the synthesis of the second-strand using DNA Polymerase I and RNase H. Exonuclease and polymerase activities were employed to convert remaining overhangs into blunt ends, after which the enzymes were removed. The 3′ ends of DNA fragments were adenylated, followed by ligation of Illumina PE adapter oligonucleotides for hybridization preparation. The AMPure XP system (Beckman Coulter) was utilized to select cDNA fragments of 400–500 bp, which were then subjected to selective enrichment in a 15-cycle PCR reaction. The resulting DNA fragments were purified using the AMPure XP system and quantified using the Bioanalyzer 2100 system (Agilent). The sequencing library was sequenced on the NovaSeq 6000 (Illumina) by Shanghai Personal Biotechnology.

Differential expression analysis was performed using HTSeq (0.9.1) to calculate the Read Count values for each gene, followed by standardization by using FPKM. Differentially expressed genes (DEGs) were identified based on an adjusted *P* value <0.05 and |log_2_fold change|>1. DEGs were then aggregated and clustered according to their expression patterns. KEGG enrichment analysis of DEGs was performed using ClusterProfiler (3.16.1) software. Protein-interaction networks based on target genes were explored using the STRING database to provide insights into the functional relationships among proteins encoded by DEGs.

Human single-nucleus RNA-seq data of aged controls and sporadic patients with AD were available from the Seattle AD Brain Cell Atlas (https://portal.brain-map.org/explore/seattle-alzheimers-disease).

### Measurement of ROS

For in vivo study, the ROS level was measured following reported procedures^19^. In brief, mice were euthanized, their brains were dissociated, frozen in liquid nitrogen and sectioned at 20 μm. The brain sections were stained with 10 μM dihydroethidium (DHE) for 30 min, followed by three washes with PBS.

For in vitro study, the ROS level was measured using DCFH-DA (S0033S, Beyotime) and DHE (S0063, Beyotime) according to the manufacturer’s instructions. In brief, BV2 cells were stimulated with 1 μg/ml PHF for 24 h with or without co-incubated with 20 μΜ YHV98-4 for treatment. The cells were then incubated with 10 μM DCFH-DA or 5 μM DHE diluted in DMEM at 37 °C in the dark for 20 min or 25 min, respectively. For the neuron–microglia coculture system, DHE was diluted in Neurobasal-A medium to 5 μM and incubated for 25 min. Intracellular ROS oxidized DCFH-DA and transformed it into fluorescent DCF. After three times washes, the fluorescence signals of DCF and DHE were measured using LSCM, with excitation/emission wavelengths set at 488/525 nm and 535/610 nm, respectively. A quantification of images was conducted on Fiji software.

### Measurement of NAD^+^ and NAD^+^/NADH level

Nicotinamide adenine dinucleotide (NAD+) and NAD^+^/nicotinamide adenine dinucleotide dehydrogenase (NADH) levels were measured using the NAD^+^/NADH Assay Kit (S0175, Beyotime) according to the manufacturer’s instructions. In brief, BV2 cells were treated with 1 μg/ml PHF with or without 20 μM YHV98-4 for 24 h. The culture medium was replaced with 200 μl per well of extraction buffer and centrifuged at 12,000*g* for 10 min at 4 °C. The supernatant was then collected. Detecting reagents were added sequentially, and the NAD_total_ level was measured at 450 nm. To measure the NADH level, the supernatant was heated at 60 °C for 30 min, followed by the addition of detecting reagents and measuring at 450 nm. The NAD^+^ level was calculated by subtracting NADH from NAD_total_.

### Measurement of MMP

Mitochondrial membrane potential (MMP) was assessed using JC-1 (C2005, Beyotime) according to the manufacturer’s instructions. In brief, following stimulation with 1 μg/ml PHF, with or without 20 μM YHV98-4, JC-1 was diluted to a final concentration of 10 μg/ml in DMEM for BV2 cells or Neurobasal-A medium for the neuron–microglia coculture system, and cells were then incubated at 37 °C for 20 min in the dark. The fluorescence signal was measured by LSCM. The excitation wavelength was 488 nm for monomers and 561 nm for aggregates, with the emission wavelength set at 530 nm and 600 nm. The red/green ratio was evaluated using ImageJ as previously reported^27^. Quantification of images was conducted on Fiji software.

### Mitochondria transfer experiments

Mitochondria transfer experiments were conducted according to the reported procedure with a few changes^13,31^. In brief, primary neurons were labeled with 20 mM MitoTracker Green (40742ES50, Yeasen) for 30 min or 1 μM DiO (40725ES10, Yeasen) for 15 min, whereas primary microglia were labeled with 50 nM MitoTracker Red CMXRos (40741ES50, Yeasen) for 30 min. For immunofluorescence staining, microglia were labeled with 200 nM MitoTracker Deep Red FM (40743ES50, Yeasen) for 30 min. After rinsing with Neurobasal-A medium three times, microglia (DIV10) were plated onto neurons (DIV10) at a ratio of 1:7 and cocultured in Neurobasal-A medium (10888022, Gibco) supplemented with 2% B-27 (17504022, Gibco), 1% penicillin–streptomycin (C0222, Beyotime) and 1% glutamax (35050061, Gibco), added with 1 μg/ml PHF with or without 20 μM YHV98-4 for 22 h.

For live-cell real-time imaging, neurons were labeled with 250 nM PK Mito Red (PKMR-2, GenVivo) for 15 min, and primary microglia were labeled with 250 nM PK Mito Deep Red (PKMDR-2, GenVivo) for 15 min. After rinsing three times with Neurobasal-A medium, microglia (DIV10) were seeded onto neurons (DIV10) at a ratio of 1:7, followed by the addition of 1 μg/ml PHF and 20 μM YHV98-4. Time-lapse imaging was initiated immediately using a Nikon AX R confocal microscope and images were acquired every 8 min.

### Measurement of mitochondrial ROS

The mitochondrial ROS level was measured using the MitoSOX Red Mitochondrial Superoxide Indicator (40778ES50, Yeasen). In brief, BV2 cells were stimulated with 1 μg/ml PHF for 24 h with or without co-incubation with 20 μM YHV98-4. The culture medium was replaced with 5 µM mitoSOX, and the cells were co-incubated for 10 min at 37 °C in the dark. After three rinses with PBS, the fluorescence signal was measured by LSCM (Leica, TCS SP8), with excitation at 510 nm and emission at 580 nm. Quantification of images was conducted using Fiji software.

For the analysis of reverse electron transport (RET), BV2 cells were plated in a 96-well black clear-bottom plate following the reported procedures^32^. A total of 18 h after plating, cells were treated with 1 μg/ml PHF with or without 20 μΜ YHV98-4 co-incubation. A total of 24 h after stimulation, the medium was replaced with RET solution consisting of 2 μM oligomycin (T21494, TargetMol) and 20 mM dimethyl succinate (112755, Sigma-Aldrich). After incubating at 37 °C for 15 min, the medium was replaced with 2 μΜ MitoSOX, either alone or in combination with 1 μM Rotenone (T2970, TargetMol) or 20 μM YHV98-4 for 30 min and rinsed with PBS. The fluorescence was measured by the microplate reader (Thermo Scientific), with excitation at 510 nm and emission at 580 nm. To analyze the RET using flow cytometry, BV2 cells were plated in a 24-well plate following the above procedures. Finally, BV2 cells were detached, centrifugated and resuspended in PBS, then detected by Attune NxT flow cytometer (Thermo Scientific).

### Measurement of mitochondrial complex activity and mitochondrial ATP level

Mitochondria were extracted using the commercial mitochondrial extraction kit (Abbkine) according to the manufacturer’s protocol. In brief, 5 × 10^6^ cells were collected, resuspended in lysis buffer and homogenized at 4 °C. Homogenization was halted when trypan blue staining indicated that 50% of the cells were positive. The sample was then centrifuged at 600*g* for 5 min at 4 °C. The resulting supernatant was further centrifuged at 11,000*g* for 10 min at 4 °C. The pellet containing the extracted mitochondria was subsequently used to detect complex activity or mitochondrial ATP levels.

Complex I activity was measured by Micro Mitochondrial Complex I Activity Assay Kit (KTB1850, Abbkine) according to the manufacturer’s protocol. In brief, the extracted mitochondria were resuspended in extracted reagents, and then the working reagent was added. The activity was detected using a microplate reader (Thermo Scientific) at 340 nm. The activity of complex I in the control group was normalized to 1, and relative values were calculated for the other groups.

Complex V activity was detected using the Micro Mitochondrial Complex V Activity Assay Kit (KTB1890, Abbkine). The extracted mitochondria were resuspended in extraction reagents, followed by the addition of enzymatic reaction reagents and phosphorus quantitative agents according to the manufacturer’s protocol. Absorbance was read at 660 nm. The activity of complex V in the control group was normalized to 1, and the relative values were calculated for the other groups.

To measure mitochondrial ATP levels, the extracted mitochondria were detected using the enhanced ATP Assay Kit (S0027, Beyotime). In brief, the mitochondrial pellet was resuspended in 100 μl of lysis buffer and centrifuged at 12,000*g* for 5 min at 4 °C. The supernatant was added to the ATP detecting buffer, and the luminescence was measured using a microplate reader (Thermo Scientific). The mitochondrial ATP value in the control group was normalized to 1.

### Measurement of mtDNA leakage

Picogreen dsDNA Quantitation Reagent (12641ES01, Yeasen) was used to stain mtDNA, and PK Mito Deep Red (PKMDR-2, GenVivo) was used to stain mitochondria. In brief, BV2 cells were stimulated with 1 μg/ml PHF for 24 h, with or without 20 μΜ YHV98-4 co-incubation. The medium was substituted with Picogreen (1:200) and incubated at 37 °C for 10 min in the dark. After three rinses, the cells were incubated with 250 nM K Mito Deep Red at 37 °C for 15 min in the dark. Fluorescence signals were acquired using a Leica TCS Stellaris8 STED microscope (Leica). The excitation and emission wavelengths were 488/520 nm for Picogreen and 647/670 nm for PK Mito Deep Red. Cytosolic mtDNA was identified by the Picogreen staining that did not colocalize with either nucleus or mitochondria^33^. Quantification of images was conducted using Fiji software.

### Analysis of autophagic flux

Autophagic flux was monitored in BV2 cells using tandem mRFP–GFP-tagged LC3 plasmids (Addgene)^34^. BV2 cells were transfected with tandem mRFP–GFP-tagged LC3 plasmids for 48 h, then treated with 1 μg/ml PHF with or without 20 μM YHV98-4 for an additional 24 h. Yellow puncta, indicating early autophagosomes, were observed due to the merging of GFP and mRFP signals, whereas red puncta (mRFP signal alone) represented late autolysosomes. Autophagic flux was evaluated by the color change of GFP–mRFP. Yellow puncta and red puncta were visualized using LSCM (Leica, TCS SP8) and quantified using Fiji software^35^.

### Analysis of mitophagic flux

pADV-mCMV-mt-mkeima (Obio Technology) was utilized to analyze the cellular mitophagic flux following the manufacturer’s instructions. In brief, BV2 cells were transduced with the adenovirus (MOI ratio of 30:1) and cultured for 24 h. Subsequently, the cells were treated with 1 μg/ml PHF with or without 20 μM YHV98-4 for an additional 24 h. Mitophagic flux was measured by quantifying the ratio of the red signal area (representing acidic mitolysosomes) to the green signal area (indicating neutral total mitochondria) using LSCM (Leica, TCS SP8) and quantified using Fiji software^33,36^.

### Phagocytosis assay

The phagocytosis capacity of BV2 cells was assessed using a beads uptake assay^37^. In brief, BV2 cells were treated with 1 μg/ml PHF with or without 20 μM YHV98-4 for 24 h. Amine-modified polystyrene latex beads (L2778, Sigma-Aldrich) were added for 4 h. After three washes, cells were fixed with 4% paraformaldehyde for 10 min. Phagocytosis of the beads by BV2 cells was visualized using LSCM (Leica, TCS SP8), and a data analysis was performed using Fiji software.

### Statistics

Statistical analysis was conducted by GraphPad Prism 9.0 (GraphPad Software). Two-tailed unpaired Student’s *t*-tests were chosen for comparisons between two groups and one-way analysis of variance (ANOVA), followed by Tukey’s post hoc analysis, was chosen for comparisons among more than two groups. For the MWM test, two-way ANOVA followed by Bonferroni post-test was applied to compare the differences in latency. The data were presented as the mean ± standard error of the mean (s.e.m.). *P* values <0.05 were considered significant.

## Results

### Hv1 upregulation in activated microglia and the restorative role of YHV98-4 in microglial homeostasis

**Fig. 1:**
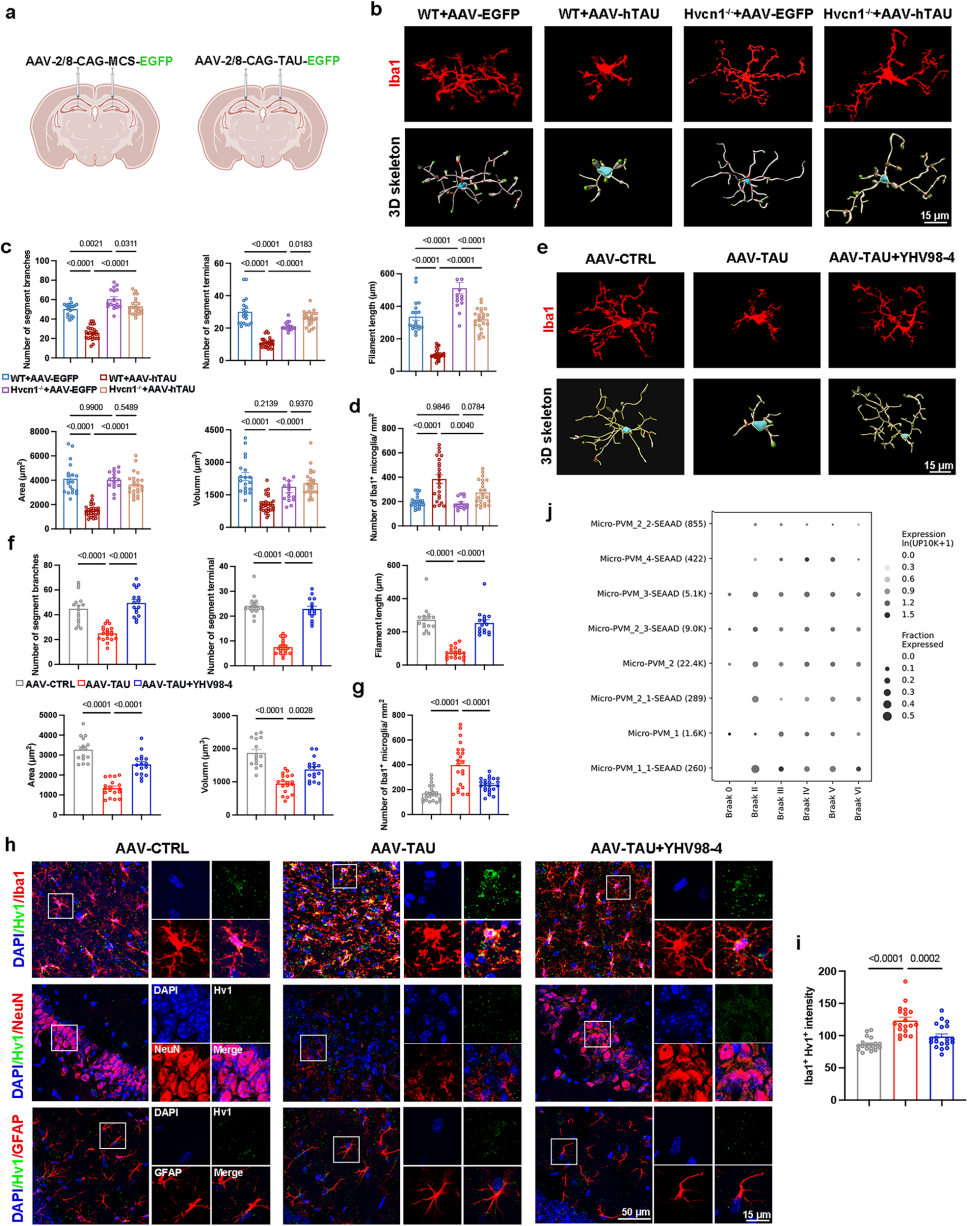
Hv1 is upregulated in activated microglia and reversed by YHV98-4. **a** A schematic representation of the intrahippocampal injection protocol. The virus constructs AAV–EGFP and AAV-hTAU were infused into the hippocampus of 2-month-old *Hvcn1*^*−/−*^ mice and littermate controls. Mice were killed 40 days post-injection for immunofluorescence analysis. **b** Representative images of Iba1 staining and 3D skeletonization in the hippocampus of *Hvcn1*^*−/−*^ mice and their littermates injected with AAV–EGFP (*Hvcn1*^*−/−*^ + AAV–EGFP and WT + AAV–EGFP) or AAV-hTAU (*Hvcn1*^*−/−*^ + AAV-hTAU and WT + AAV-hTAU). The red dots indicate branching points, and the green dots indicate terminal points. **c** A quantification of microglial branch number, terminal points and filament length using Imaris software (*n* = 16-27 cells from three to five mice). **d** A quantification of the number of Iba1^+^ microglia per square millimeter in the hippocampus (*n* = 14-23 fields of view from three to five mice). **e**,**f** Representative immunofluorescent images and corresponding 3D skeletonization of microglia (**e**) and quantification of branch number, terminal points and filament length (**f**) in the hippocampus of C57BL/6 mice injected with AAV–EGFP (AAV-CTRL), AAV-hTAU (AAV-TAU) or AAV-hTAU with YHV98-4 treatment (AAV-TAU + YHV98-4) (*n* = 16–19 cells from five mice). **g** A quantification of the number of Iba1^+^ microglia per square millimeter in the hippocampus (*n* = 22 fields of view from five mice). **h**,**i** Representative immunofluorescent images showing the colocalization of DAPI (blue), Hv1 (green) with Iba1 (red), NeuN (red) or GFAP (red) (**h**) and quantification of Hv1 intensity (**i**) (*n* = 19 fields of view from five mice). **j** *Hvcn1* expression in different microglial subclusters from patients with AD with varying Braak stages. The data were calculated using a one-way ANOVA followed by a Tukey’s post hoc analysis and presented as the mean ± s.e.m. The *P* values are presented on the graph.

Hv1 is an essential ion channel involved in ROS production and oxidative stress, both of which are strongly linked to AD pathology^14^. Given this connection, we investigate the role of Hv1 in AD by performing intrahippocampal stereotaxic injection of AAVs containing human tau (AAV2/8-CAG-TAU-EGFP-3FLAG-WPRE, AAV-hTAU) or a control virus vector (AAV2/8-CAG-MCS-EGFP-3FLAG, AAV–EGFP) in male *Hvcn1* knockout (*Hvcn1*^*−/−*^*)* mice and their littermate controls (Fig. 1a and Supplementary Fig. 1a). A total of 28 days post-injection, we assessed microglial morphologies through 3D reconstruction and Imaris quantification. In WT mice injected with AAV-hTAU (WT + AAV-hTAU), microglia exhibited fewer terminal and branch points, as well as decreases in filament length, surface area and volume compared with those injected with AAV–EGFP (WT + AAV–EGFP) (Fig. 1b,c). The morphological changes indicate microglial activation after injecting AAV-hTAU^38^. In addition, increased microglial proliferation in WT + AAV-hTAU mice (Fig. 1d) further supports the transition from a homeostatic to an activated state in tau pathology^38^. To further examine the role of Hv1 in this process, we generated *Hvcn1*^*−/−*^ mice as previously reported^19^ and injected them with AAV–EGFP (*Hvcn1*^*−/−*^ + AAV–EGFP) or AAV-hTAU (*Hvcn1*^*−/−*^ + AAV-hTAU). Significantly, microglia in *Hvcn1*^*−/−*^ + AAV-hTAU mice exhibited a reversion to a steady-state morphology, indicating that Hv1 deficiency mitigates tau-induced microglial activation (Fig. 1b,c).

Our previous study identified YHV98-4 as a selective Hv1 channel inhibitor with confirmed blood-brain barrier permeability^19^. To assess its therapeutic potential, C57BL/6 mice injected with AAV-hTAU received intrahippocampal administration of YHV98-4 (10 mg/kg daily) for 21 days (AAV-TAU + YHV98-4) (Supplementary Fig. 1b,c). No significant adverse effects were observed during the treatment period (Supplementary Fig. 1d). YHV98-4 treatment reversed microglial morphological changes and proliferation, restoring microglial homeostasis (Fig. 1e–g). Consistent with previous studies^16^, we found that Hv1 is predominantly expressed in microglia rather than in neurons or astrocytes (Fig. 1h). Hv1 expression, which increased concurrently with microglia activation in AAV-TAU mice, was significantly reduced following YHV98-4 treatment (Fig. 1h, i). Furthermore, given that CD11c is regarded as a marker of disease-associated microglia (DAM), and that MHC II^+^ microglia play a pivotal role in regulating neuroinflammation via mediating the interaction between innate immunity and T cell-mediated adaptive immunity^3,39^, we analyzed their expression and showed a strong correlation among CD11c^+^ microglial subsets, MHC II^+^ microglial subsets and Hv1 (Supplementary Fig. 2).

Given that Braak stage correlates with tau pathology and is driven by microglial activation^40^, we further analyzed data from the published Seattle Alzheimer’s Disease Brain Cell Atlas, specifically the middle temporal gyrus taxonomy, in which microglia and perivascular macrophages (micro-PVM) from patients with AD were classified into several supertypes^41^. Compared with Braak stage 0, both the fraction and intensity of Hv1 expression in Micro-PVM increased from Braak stages II to VI, particularly in subtypes identified as reacting and enhanced redox (Micro-PVM_2.1 and Micro-PVM_2.3), DAM (Micro-PVM_3) and lipid-associated subtype (Micro-PVM_4)^41,42^ (Fig. 1j). These findings suggest that Hv1 is upregulated in activated microglia in AD, whereas YHV98-4 treatment restores microglial homeostasis and concomitantly reduces Hv1 expression.

### Hv1 regulates tau spreading and neuroinflammation

**Fig. 2:**
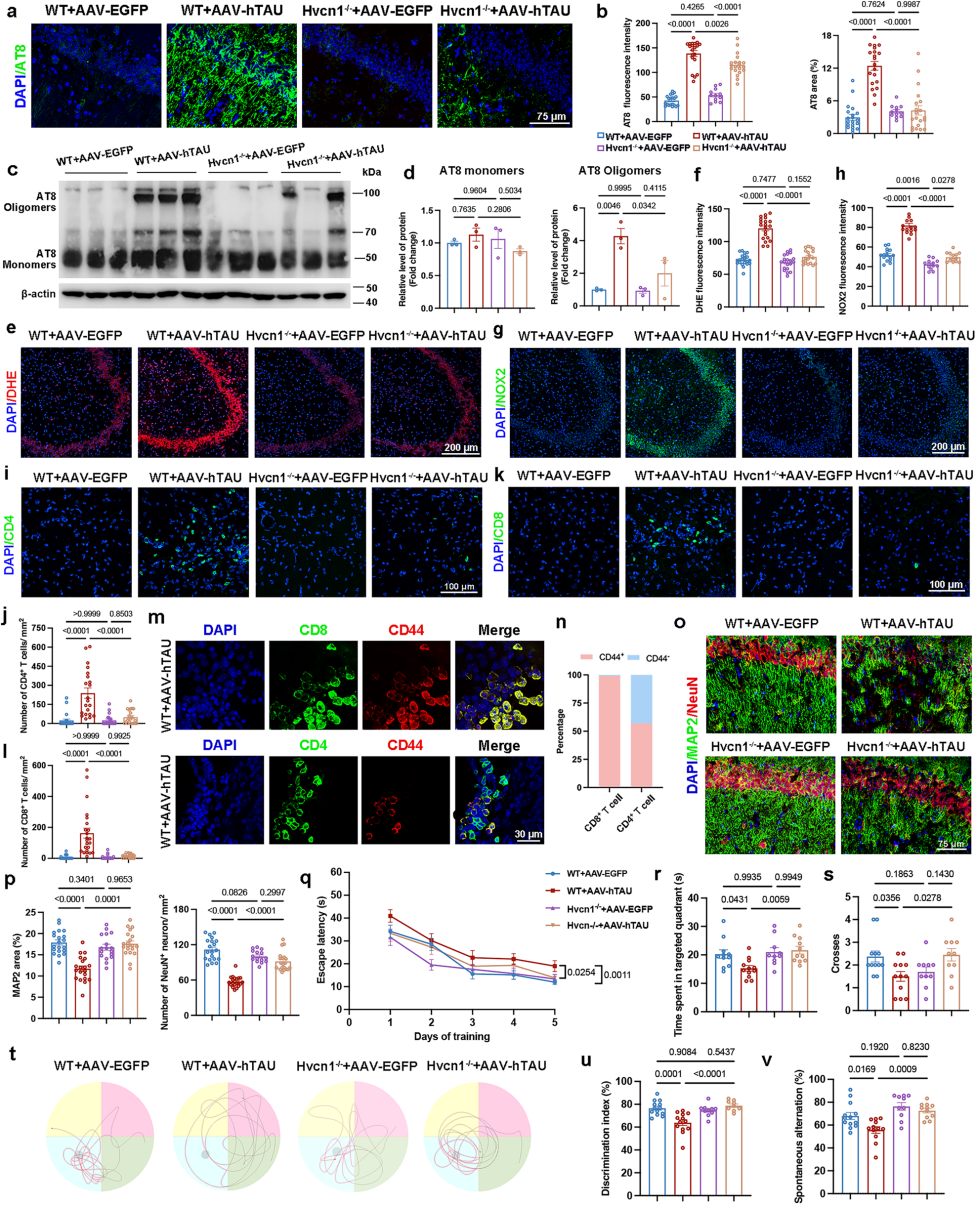
*Hvcn1* deletion alleviates tau pathology and tau-induced neuroinflammation. **a** Representative immunofluorescence staining of p-Tau detected by AT8 (green) colabeled with DAPI (blue) in WT + AAV–EGFP, WT + AAV-hTAU, *Hvcn1*^*−/−*^ + AAV–EGFP and *Hvcn1*^*−/−*^ + AAV-hTAU mice. **b** A quantification of AT8 coverage and intensity in the hippocampus (*n* = 12–20 fields of view from three to five mice). **c** Western blot images showing AT8 oligomers and monomers from hippocampal lysates. **d** A quantification of AT8 monomer levels and AT8 oligomer levels in the hippocampus (*n* = 3). **e**,**f** Representative images of DHE staining in the hippocampus (**e**) and quantification of DHE fluorescence intensity (**f**) (*n* = 19-21 fields of view from five mice). **g**, **h** Representative images of NOX2 staining in the hippocampus (**g**) and quantification of NOX2 fluorescence intensity (**h**) (*n* = 13–15 fields of view from three mice). **i**–**l** Representative confocal images and quantification of CD4⁺ (**i** and **j**) and CD8⁺ (**k** and **l**) T cells (green) in the hippocampus, quantifying the number of cells per square millimeter (*n* = 21 fields of view from five mice for CD4⁺ T cells; *n* = 19–23 fields of view from five mice for CD8⁺ T cells). **m**,**n** Representative confocal images of CD44 (red) colabeled with CD8 (green) or CD4 (green) (**m**) and quantification of the proportion of CD44^+^CD8^+^ T cells and CD44^+^CD4^+^ T cells (*n*). **o**,**p** Representative images of NeuN (green) colabeled with MAP2 (red) and DAPI (blue) (**o**) and quantification of MAP2 coverage and NeuN intensity in the hippocampus of WT + AAV–EGFP, WT + AAV-hTAU, *Hvcn1*^*−/−*^ + AAV–EGFP and *Hvcn1*^*−/−*^ + AAV-hTAU mice (**p**) (*n* = 15-20 fields of view from three to five mice). **q**–**t** MWM test of WT + AAV–EGFP (*n* = 12), WT + AAV-hTAU (*n* = 12), *Hvcn1*^*−/−*^ + AAV–EGFP (*n* = 10) and *Hvcn1*^*−/−*^ + AAV-hTAU (*n* = 11) mice were evaluated. Escape latency in the training trial (**q**), duration in the target quadrant (**r**) and quantification of the platform crossings (**s**), and representative track paths during the probe trials (**t**). **u**,**v** A quantification of discrimination index percentage in a NOR test (**u**) and spontaneous alternation percentage in a Y-maze test (**v**) of WT + AAV–EGFP (*n* = 12), WT + AAV-hTAU (*n* = 12), *Hvcn1*^*−/−*^ + AAV–EGFP (*n* = 10) and *Hvcn1*^−^^/*−*^ + AAV-hTAU (*n* = 11). The data were calculated using a one-way ANOVA followed by a Tukey’s post hoc analysis and presented as the mean ± s.e.m. The *P* values are presented on the graph.

We then explored the functional role of Hv1 in AD pathogenesis using *Hvcn1*^*−/−*^ mice. Following intrahippocampal injection of AAV-hTAU, immunofluorescence analysis of AT8 (which targets phosphorylated tau (p-tau) at Ser202/Thr205 epitopes) demonstrated a marked reduction in AT8 expression and distribution in *Hvcn1*^*−/−*^ mice compared with littermate controls (Fig. 2a,b and Supplementary Fig. 1a). Western blot analysis was also performed to detect oligomeric (75–150 kDa) and monomeric (50–65 kDa) forms of AT8^43^. Notably, AT8 oligomers, which are associated with synaptotoxicity^43,44^, were increased in WT + AAV-hTAU mice but attenuated in *Ηvcn1*^*−/−*^ + AAV-hTAU mice (Fig. 2c,d).

Neuroinflammation is a key feature of AD^2^. A well-known function of Hv1 is its regulation of NOX2-mediated ROS production, which contributes to neuroinflammation^16,45^. Using DHE staining, we found that tau-induced ROS elevation was reversed in *Hvcn1*^*−/−*^ + AAV-hTAU mice (Fig. 2e,f). Moreover, NOX2 levels were significantly decreased in *Hvcn1*^*−/−*^ + AAV-hTAU mice compared with WT + AAV-hTAU mice (Fig. 2g,h). A principal component analysis showed distinct clustering between *Hvcn1*^*−/−*^ + AAV-hTAU and WT + AAV-hTAU mice brains (Supplementary Fig. 3a). Importantly, gene set enrichment analysis (GSEA) showed a significant downregulation of adaptive and innate immune response pathways, as well as proinflammatory cytokine production, in *Hvcn1*^*−/−*^ + AAV-hTAU mice (Supplementary Fig. 3b). Transcriptional analysis further identified a substantial reduction in DEGs involved in antigen presentation (Supplementary Fig. 3c). Moreover, type 1 IFN signaling, which is known to be stimulated by tau and implicated in exacerbating AD pathology and cognitive deficits^9^, was suppressed in *Hvcn1*^*−/−*^+AAV-hTAU mice (Supplementary Fig. 3 d). Protein-interaction network analysis based on DEGs highlighted the association of Hv1 with other immune response-related proteins (Supplementary Fig. 3e). Recent studies have emphasized the detrimental immune niches shaped by interactions between microglia-mediated innate immune responses and T cell-mediated adaptive immunity. T cell infiltration and activation within the brain are directly linked to cytotoxicity, significantly contributing to neuroinflammation and neurodegeneration^3,46,47^. In our study, hippocampal infiltration of CD4^+^ and CD8^+^ T cells was markedly increased in tau pathology (Fig. 2i–l). Furthermore, CD44, an established marker of effector and memory T cells that infiltrate in the CNS^48,49^, was expressed in nearly 100% of hippocampal-infiltrating CD8^+^ T cells and 57% of CD4^+^ T cells (Fig. 2m,n). Remarkably, *Hvcn1*^*−/−*^ efficiently blocked the tau-induced infiltration of both CD4^+^ and CD8^+^ T cells (Fig. 2i–l).

Lastly, we investigated whether Hv1 contributes to neuronal damage and cognitive dysfunction in tauopathy mice. Immunofluorescence costaining of NeuN and MAP2 revealed that *Hvcn1* deletion prevented synaptic damage and rescued neuronal loss (Fig. 2o,p). To assess cognitive function, we performed the MWM test and demonstrated that there were no cognitive differences between WT + AAV–EGFP and *Hvcn1*^*−/−*^ + AAV–EGFP mice. However, in mice receiving hippocampal injection of AAV-hTAU, *Hvcn1* deletion significantly rescued tau-induced cognitive impairments (Fig. 2q–t). Specifically, *Hvcn1*^−/−^ + AAV-TAU mice spent less time locating the hidden platform during the training period (Fig. 2q), exhibited more platform crossings (Fig. 2r), spent more time in the target quadrant during the probe trial (Fig. 2s) and displayed more purposeful swimming patterns (Fig. 2t). Moreover, compared with WT + AAV-hTAU mice, *Hvcn1*^−/−^+AAV-hTAU mice spent significantly more time exploring the novel object in the NOR test (Fig. 2u) and were more likely to remember previously visited arms and explore the novel arm in the Y-maze test (Fig. 2v).

Together, these results demonstrated that microglial Hv1 plays an important role in hyperphosphorylated tau propagation, neuroinflammation, neuronal health, and cognitive function.

### YHV98-4 alleviates tau pathology and tau-induced neuroinflammation

**Fig. 3:**
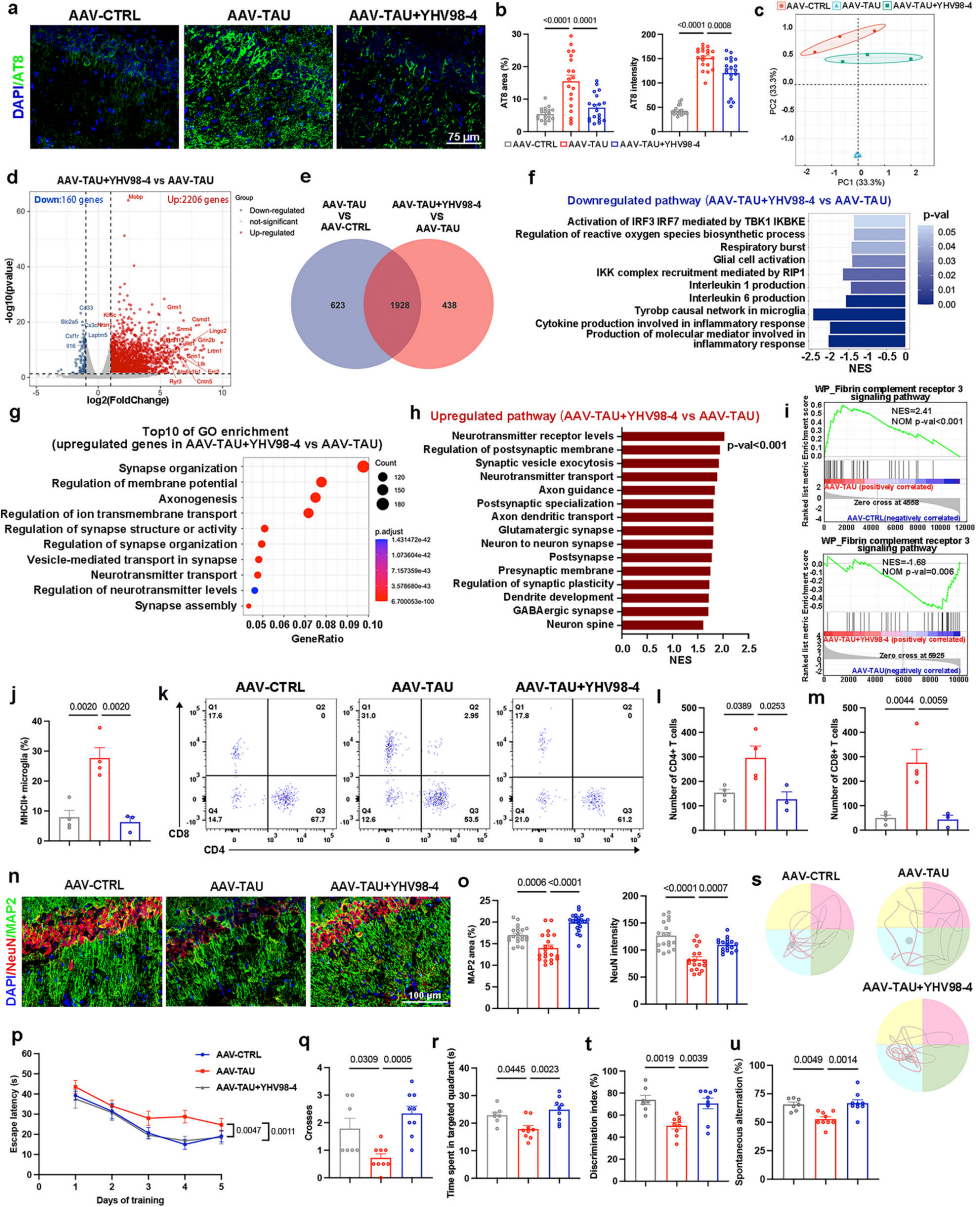
Inhibiting Hv1 mitigates tau spreading and attenuates neuroinflammation in tauopathy mice. **a** Representative immunofluorescence staining of p-Tau detected by AT8 (green) with DAPI (blue). **b** A quantification of AT8 coverage and AT8 intensity in the hippocampus of AAV-CTRL, AAV-TAU and AAV-TAU + YHV98-4 (*n* = 19 fields of view from five mice). **c** Principal component analysis of microglial RNA-seq data from AAV-CTRL, AAV-TAU and AAV-TAU + YHV98-4 groups (*n* = 3 mice per group). **d** Volcano plots of DEGs in AAV-TAU + YHV98-4 versus AAV-TAU groups. The red dots indicate upregulated genes, the blue dots indicate downregulated genes and the gray dots represent nonsignificant genes. Selected genes are labeled. DEGs were identified using an adjusted *P* value <0.05 and |log_2_fold change| >1. **e** Venn diagram of DEGs in AAV-TAU versus AAV-CTRL and AAV-TAU + YHV98-4 versus AAV-TAU comparisons. The intersection represents common DEGs between the two groups. **f** A GSEA of major inflammation-related pathways downregulated in AAV-TAU + YHV98-4 versus AAV-TAU. **g** Top ten GO enrichment terms of upregulated DEGs in AAV-TAU + YHV98-4 versus AAV-TAU. **h** A GSEA of significantly upregulated pathways enriched in AAV-TAU + YHV98-4 versus AAV-TAU (*P* < 0.001) based on microglial RNA-seq data. **i** GSEA plots depicting enrichment of the ‘Fibrin complement receptor 3 signaling pathway’ in AAV-TAU versus AAV-CTRL and in AAV-TAU + YHV98-4 versus AAV-TAU. **j** A quantification of MHC II-positive microglia in the hippocampus and cortex of AAV-CTRL (*n* = 4), AAV-TAU (*n* = 3) and AAV-TAU + YHV98-4 (*n* = 3) mice. **k** Flow cytometry plots showing distinct T cell populations identified by CD4 and CD8. **l**,**m** A quantification of CD4^+^ T cell (Q3) (**l**) and CD8^+^ T cell (Q1) number (**m**) in the hippocampus and cortex of AAV-CTRL (*n* = 4), AAV-TAU (*n* = 3) and AAV-TAU + YHV98-4 (*n* = 3) mice. **n**,**o** Representative images of mouse hippocampus sections stained with NeuN (red) and MAP2 (green) (**n**) and quantification of MAP2 coverage and NeuN intensity (**o**) in the hippocampus of AAV-CTRL, AAV-TAU and AAV-TAU + YHV98-4 mice (*n* = 21 fields of view from five mice). **p**–**s** Behavioral assessments in AAV-CTRL (*n* = 7), AAV-TAU (*n* = 9) and AAV-TAU + YHV98-4 (*n* = 9) mice, including escape latency during the training trails of the MWM test (**p**), quantification of platform crossings (**q**) and time spent in the target quadrant (**r**), and representative track paths (**s**) during the probe trials. **t**,**u** A quantification of the discrimination index in the NOR test (**t**) and the percentage of spontaneous alternation in the Y-maze test (**u**). The data were calculated using a one-way ANOVA followed by a Tukey’s post hoc analysis and presented as the mean ± s.e.m. The *P* values are presented on the graph.

To validate the therapeutic potential of YHV98-4 in AD pathology, we examined its effects on tau propagation and neuroinflammation. YHV98-4 significantly reduced the propagation of p-tau, as evidenced by AT8 staining (Fig. 3a,b and Supplementary Fig. 1b). However, no significant reduction in oligomeric p-tau levels in the hippocampus was observed following YHV98-4 treatment (Supplementary Fig. 4a, b).

To further support the protective role of YHV98-4 and elucidate its underlying mechanism, we isolated microglia from the hippocampus and cortex and performed transcriptome sequencing. Both principal component analysis and cluster analysis showed that AAV-CTRL and AAV-TAU + YHV98-4 were highly distinct from AAV-TAU, while no clear separation was observed between AAV-CTRL and AAV-TAU + YHV98-4 (Fig. 3c and Supplementary Fig. 4c). Further analysis showed that 2,359 genes were downregulated and 192 genes were upregulated in AAV-TAU compared with AAV-CTRL (Supplementary Fig. 4d). After YHV98-4 treatment, 2,206 genes were upregulated and 160 genes were downregulated (Fig. 3d), with 1928 genes overlapping, suggesting that 75.6% of tau-induced transcriptional changes were restored following YHV98-4 treatment (Fig. 3e). GSEA revealed the ‘Tyrobp causal network in microglia’ as the most significantly enriched pathway in AAV-TAU compared with AAV-CTRL, which was markedly downregulated after YHV98-4 treatment (Supplementary Fig. 4e). Heat map analysis revealed a marked downregulation of DAM-related genes in AAV-TAU mice compared with AAV-CTRL, whereas treatment with YHV98-4 significantly increased their expression (Supplementary Fig. 4f). In addition, GSEA revealed that numerous inflammation-related pathways, especially those regulating cytokine and ROS production that were upregulated by tau (Supplementary Fig. 4g), were downregulated following YHV98-4 treatment (Fig. 3f). Furthermore, both GO enrichment and GSEA analyses revealed that while microglia in AAV-TAU mice actively contribute to synaptic damage by disrupting synapse organization, assembly, plasticity and neurotransmitter transport (Supplementary Fig. 4h,i), YHV98-4 treatment substantially reversed these pathological pathways, thereby mitigating microglia-driven neurotoxicity in tauopathy (Fig. 3g,h). In addition, GSEA revealed significant enrichment of the ‘fibrin complement receptor 3 signaling pathway’ in AAV-TAU mice compared with AAV-CTRL, which was downregulated after YHV98-4 treatment (Fig. 3i).

To validate these findings, flow cytometry was employed to measure microglia, mononuclear macrophages and lymphocytes in the mouse brain parenchyma (Supplementary Fig. 4j). We sorted MHC II^+^ microglia and revealed that YHV98-4 decreased the percentage of MHC II^+^ microglia in tauopathy (Fig. 3j and Supplementary Fig. 4k). Correspondingly, tau-induced infiltration of both CD4^+^ and CD8^+^ T cells was decreased following YHV98-4 treatment (Fig. 3k–m). Notably, no significant alterations were observed in the number or MHC II^+^ proportion of mononuclear macrophages (Supplementary Fig. 4l,m). Immunofluorescence analysis further confirmed that YHV98-4 successfully prevented tau-induced CD4^+^ and CD8^+^ T cell infiltration in the hippocampus (Supplementary Fig. 4n–q). Moreover, we examined the role of YHV98-4 in protecting neurons and cognitive function. Tau-induced synaptic damage and neuronal loss in AAV-TAU mice were significantly reversed following YHV98-4 treatment (Fig. 3n,o). In the MWM (Fig. 3p–s), NOR (Fig. 3t) and Y-maze tests (Fig. 3u), AAV-TAU + YHV98-4 mice showed significantly improvements in memory, learning and overall cognitive function compared with AAV-TAU mice (Fig. 3p–u). Overall, these findings underscore that YHV98-4 effectively prevents tau hyperphosphorylation and spreading, restores both innate and adaptive immune homeostasis, protects neurons and rescues cognitive impairments in tauopathy.

### Inhibiting Hv1 attenuates AD pathology and cognitive impairments in 3×Tg AD mice

**Fig. 4:**
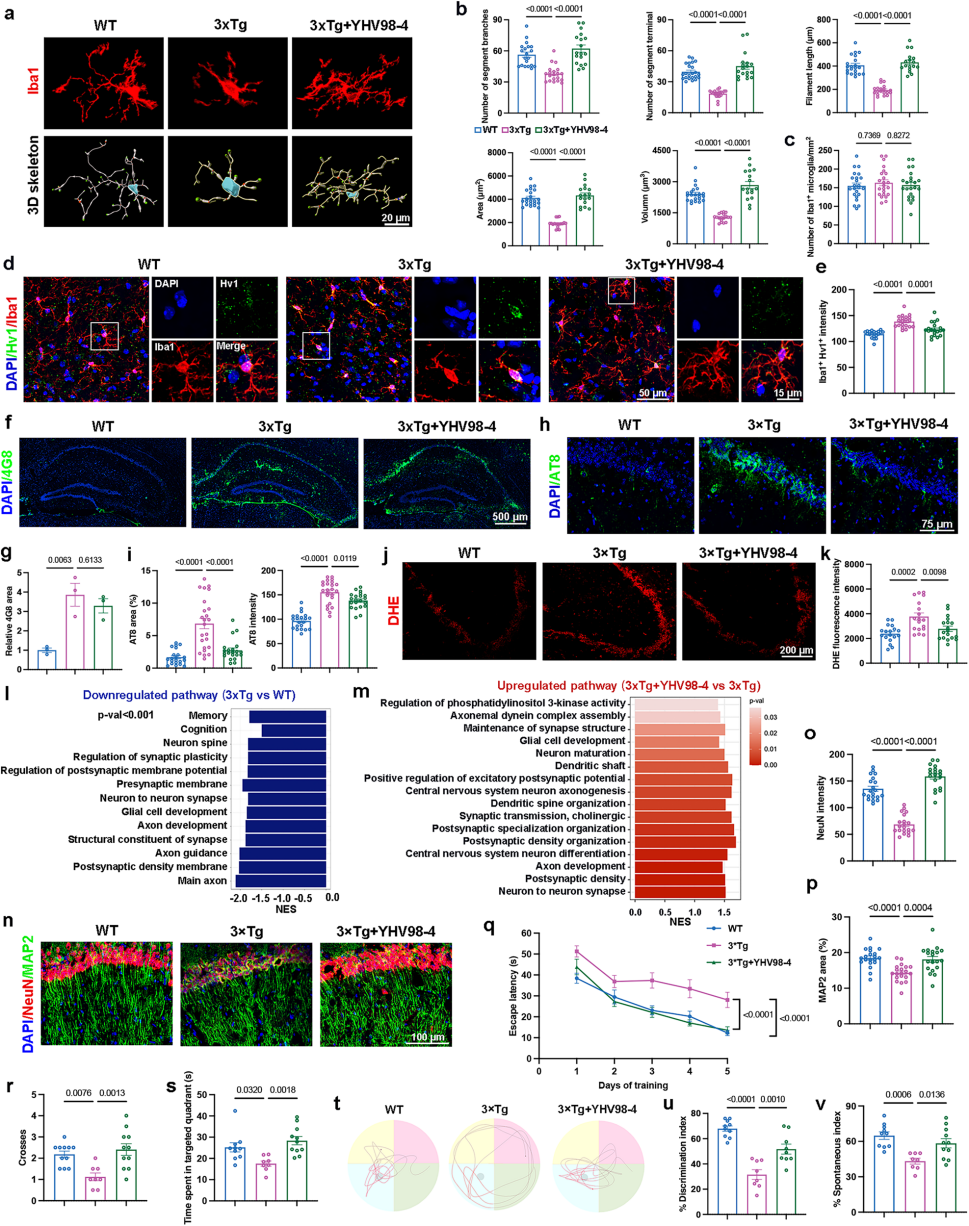
Inhibiting Hv1 restores microglial homeostasis, alleviates tau pathology and neuroinflammation and improves cognitive function in 3×Tg AD mice. **a–c** Representative staining of Iba1^+^ microglia and corresponding 3D skeletonization (**a**), quantification of microglial morphology (**b**) (*n* = 18–23 cells from five mice) and microglial number (**c**) (*n* = 20–23 fields of view from five mice) in WT, 3×Tg and 3×Tg+YHV98-4 mice. **d**,**e** Representative images showing Hv1 (green) costained with Iba1 (red) and DAPI (blue) (**d**) and quantification of Hv1 intensity (**e**) in the hippocampus (*n* = 20 fields of view from five mice). **f**,**g** Representative images of 4G8 staining in the hippocampus (**f**) and quantification of 4G8^+^ area (**g**) (*n* = 3 mice). **h**,**i** Representative AT8 staining (**h**) and quantification of AT8 coverage and intensity (**i**) in the hippocampus (*n* = 21–24 fields of view from five mice). **j**,**k** Representative images of DHE staining in the hippocampus (**j**) and quantification of DHE fluorescence intensity (**k**) (*n* = 18 fields of view from three mice). **l** GSEA of significant downregulated pathways in microglia of 3×Tg versus WT mice. **m** GSEA of significantly upregulated pathways in microglia of 3×Tg+YHV98-4 versus 3×Tg mice. **n**–**p** Representative images of NeuN and MAP2 staining (**n**) and quantification of NeuN intensity (**o**) and MAP2 coverage (**p**) (*n* = 19–20 fields of view from five mice). **q**–**v** Behavioral assessments in WT (*n* = 10), 3×Tg (*n* = 8) and 3×Tg+YHV98-4 mice (*n* = 11) mice. Escape latency during MWM training trials (**q**), quantification of platform crossings (**r**) and time spent in the target quadrant (**s**), and representative track paths during the probe trials (**t**). A quantification of the discrimination index in the NOR test (**u**) and the percentage of spontaneous alternation in the Y-maze test (**v**). The data were calculated using a one-way ANOVA followed by a Tukey’s post hoc test in **b**, **c**, **e**, **g**, **i**, **k**, **o**, **p**, **r**, **s**, **u** and **v** and a two-way ANOVA followed by a Bonferroni post hoc test in **q**. The data are presented as the mean ± s.e.m. The *P* values are presented on the graph.

We further validated the protective potential of YHV98-4 in 3×Tg mice, an AD model characterized by both Aβ and tau pathology. At 7 months of age, mice were administrated YHV98-4 via intraperitoneal injection at a dose of 10 mg/kg for 1 month (3×Tg+YHV98-4), without inducing toxicity (Supplementary Fig. 5a,b). A 3D reconstruction of Iba1 staining revealed morphological alterations in microglia in 3×Tg mice, which returned to a homeostatic state following YHV98-4 treatment, without significant changes in microglial proliferation (Fig. 4a–c). Costaining of Hv1 and Iba1 showed high Hv1 expression in activated microglia in 3×Tg mice, whereas expression was reduced in 3×Tg+YHV98-4 mice (Fig. 4d,e). As 3×Tg mice display both Aβ and tau pathologies, we assessed Aβ levels using 4G8 and phosphorated tau using AT8. Immunofluorescence staining showed that YHV98-4 alleviated p-tau accumulation but had little effect on Aβ levels in 3×Tg mice (Fig. 4f–i). DHE staining showed elevated ROS levels in 3×Tg mice, which were reduced following YHV98-4 treatment (Fig. 4j,k).

Transcriptomic analysis of microglia revealed significant synaptic damage and memory-related deficiencies in 3×Tg mice, which were markedly rescued following YHV98-4 treatment, restoring neuronal functionality (Fig. 4l,m). Immunofluorescence analysis further confirmed that YHV98-4 protects neurons and prevents neuronal loss in AD mice (Fig. 4n–p). To evaluate cognitive function, we conducted behavioral tests. In the MWM test, 3×Tg mice exhibited spatial learning and memory impairments, whereas 3×Tg+YHV98-4 mice showed substantial improvements (Fig. 4q–t). Moreover, 3×Tg+YHV98-4 mice demonstrated enhanced memory performance in both the NOR test (Fig. 4u) and the Y-maze test (Fig. 4v), compared with 3×Tg mice. Overall, these findings highlight the therapeutic potential of YHV98-4 in alleviating tau pathology and neuroinflammation, preventing neuronal loss and improving cognitive function in AD.

### Inhibiting Hv1 restores microglial homeostasis by rescuing mitochondrial ETC abnormalities

**Fig. 5:**
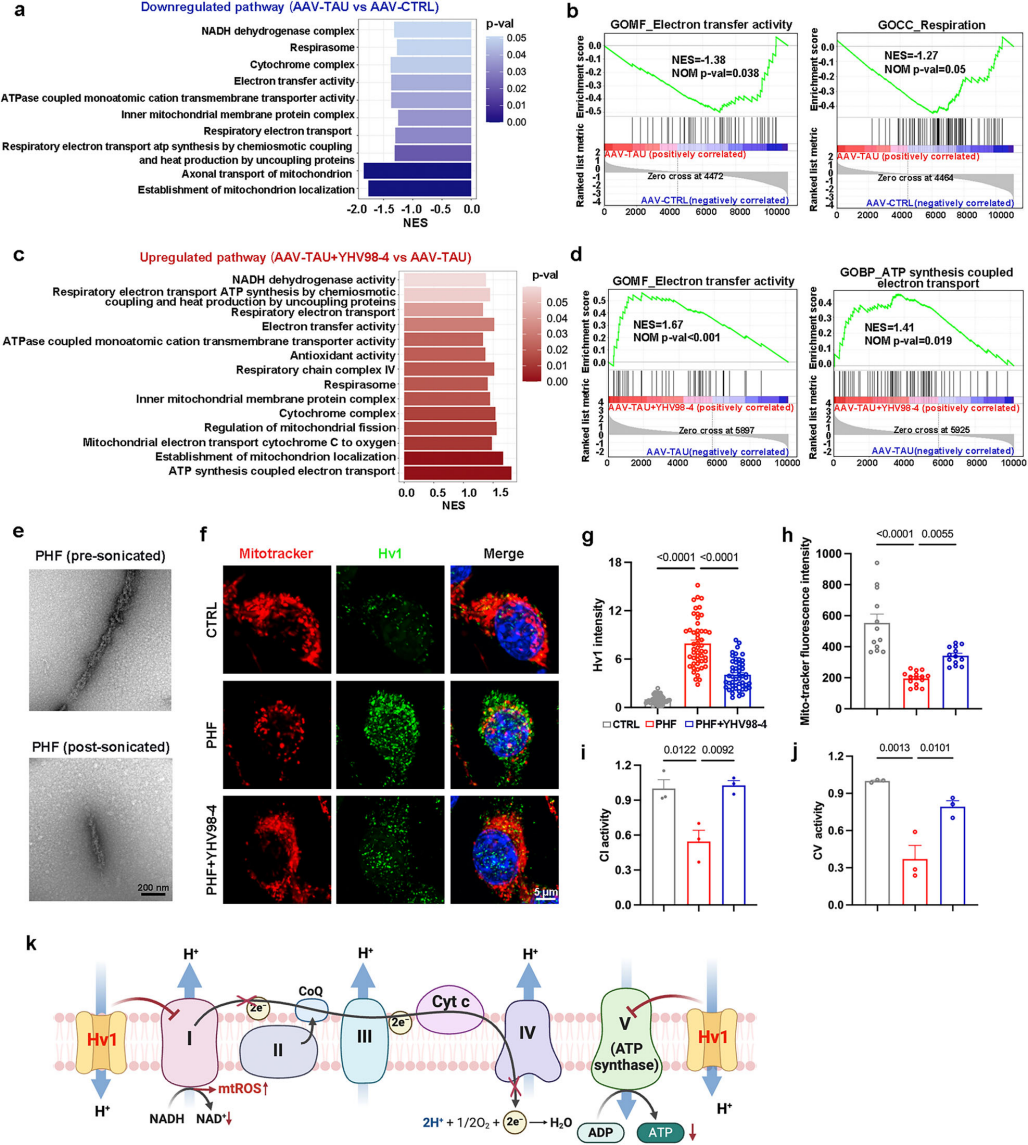
Inhibiting Hv1 restores microglial mitochondrial ETC. **a** A GSEA analysis showing significantly downregulated mitochondria-associated pathways in AAV-TAU compared with AAV-CTRL. **b** GSEA plots depicting ‘electron transport activity’ and ‘respirasome’ pathways were enriched in AAV-CTRL compared with AAV-TAU. **c** A GSEA showing significantly upregulated mitochondria-associated pathways in AAV-TAU + YHV98-4 compared with AAV-TAU. **d** GSEA plots depicting enrichment of ‘electron transport activity’ and ‘ATP synthesis coupled electron transport’ in AAV-TAU + YHV98-4 compared with AAV-TAU. **e** Representative transmission electron microscopy images of PHF before and after sonication. **f** Representative images of MitoTracker (red) colabeled with Hv1 (green) and DAPI (blue) in BV2 cells. **g**,**h** A quantification of Hv1 intensity (*n* = 48–50 cells from three independent experiments) (**g**) and MitoTracker fluorescence intensity (**h**) (*n* = 12–14 fields of view from three independent experiments) in PHF-treated cells, with or without YHV98-4 treatment. **i**,**j** Measurement of CI activity (**i**) and CV activity (**j**) in cells treated with 1 μg/ml PHF, with or without 20 μM YHV98-4 (*n* = 3 independent experiments). **k** A schematic diagram illustrating how Hv1 triggers ETC damage. CI, complex I; CV, complex V. The data were calculated using a one-way ANOVA followed by a Tukey’s post hoc analysis and presented as the mean ± s.e.m. The *P* values are presented on the graph.

To further elucidate the underlying mechanisms of microglial Hv1 in AD pathologies, we analyzed microglial transcriptomic data and found that the ETC, a crucial mitochondrial component responsible for energy production and redox homeostasis, was extensively damaged in both the AAV-TAU and 3×Tg mouse models (Fig. 5a,b and Supplementary Fig. 6a). This impairment primarily manifested as dysfunctional mitochondrial complexes, reduced electron transfer activity and decreased ATP synthesis driven by the proton motive force. Surprisingly, inhibition of Hv1 completely restored ETC function in both tauopathy and 3×Tg mice models (Fig. 5c, d and Supplementary Fig. 6b). In addition, mitochondrial biogenesis was impaired in AAV-TAU mice but was rescued by YHV98-4 treatment (Supplementary Fig. 6c,d). Given the pivotal role of the proton gradient in electron transfer and ATP synthesis, we hypothesized that Hv1 modulates mitochondrial complexes activity, thereby affecting the ETC. To investigate this hypothesis, we synthesized PHF and utilized them to stimulate BV2 cells, mimicking microglia in tauopathy (Fig. 5e). Since the presence of Hv1 in microglial mitochondria has not been previously reported, we first evaluated its colocalization with mitochondria. Hv1 expression was upregulated in PHF-treated microglia and detected in mitochondria but significantly decreased following YHV98-4 treatment (Fig. 5f,g). To further assess mitochondrial function, we measured MitoTracker fluorescence intensity, which correlates positively with mitochondrial health. The increased fluorescence intensity observed following YHV98-4 treatment suggested that Hv1 inhibition alleviates mitochondrial dysfunction (Fig. 5h).

Given that complex I is a major site of ROS production and complex V (ATP synthase) drives ATP synthesis through proton gradient coupling, we especially focused on the effects of YHV98-4 on these two mitochondrial complexes^50^. Heat map analysis showed that genes encoding Complex Ι were downregulated in tau pathology but upregulated after ΥΗV98-4 treatment (Supplementary Fig. 6e). Subsequent validation in BV2 cells confirmed decreased complex I activity following PHF stimulation, which was restored by YHV98-4 treatment (Fig. 5i). GSEA further revealed that AAV-TAU mice exhibited increased superoxide anion production, whereas ΥΗV98-4 enhanced antioxidant activity (Supplementary Fig. 6f). Moreover, YHV98-4 reversed the tau-induced suppression of genes encoding complex V (Supplementary Fig. 6g). Consistently, complexes V activity in BV2 cells was reduced by PHF stimulation but reversed upon YHV98-4 treatment (Fig. 5j). Heat map analysis also showed that oxidative phosphorylation-related genes were downregulated in AAV-TAU mice and effectively restored by YHV98-4 treatment (Supplementary Fig. 6h). We then measured hippocampal ATP levels and confirmed that YHV98-4 alleviated tau-induced energy deficits (Supplementary Fig. 6i). Furthermore, GSEA revealed enhanced ATP-dependent selective autophagy and lysosomal pH regulation in AAV-TAU + YHV98-4 mice compared with AAV-TAU mice (Supplementary Fig. 6j,k). Western blot analysis of LC3 conversion (LC3-I to LC3-II) revealed decreased LC3-II levels in AAV-TAU mice, which were restored by YHV98-4 treatment^51^ (Supplementary Fig. 6l,m). In summary, our findings confirm that Hv1 is expressed in mitochondria and plays a regulatory role in the ETC, particularly impacting complex I and complex V (Fig. 5k). Inhibiting Hv1 with YHV98-4 effectively restores mitochondrial ETC function and electron transfer activity.

### Inhibiting Hv1 ameliorates microglial mitochondrial dysfunction

**Fig. 6:**
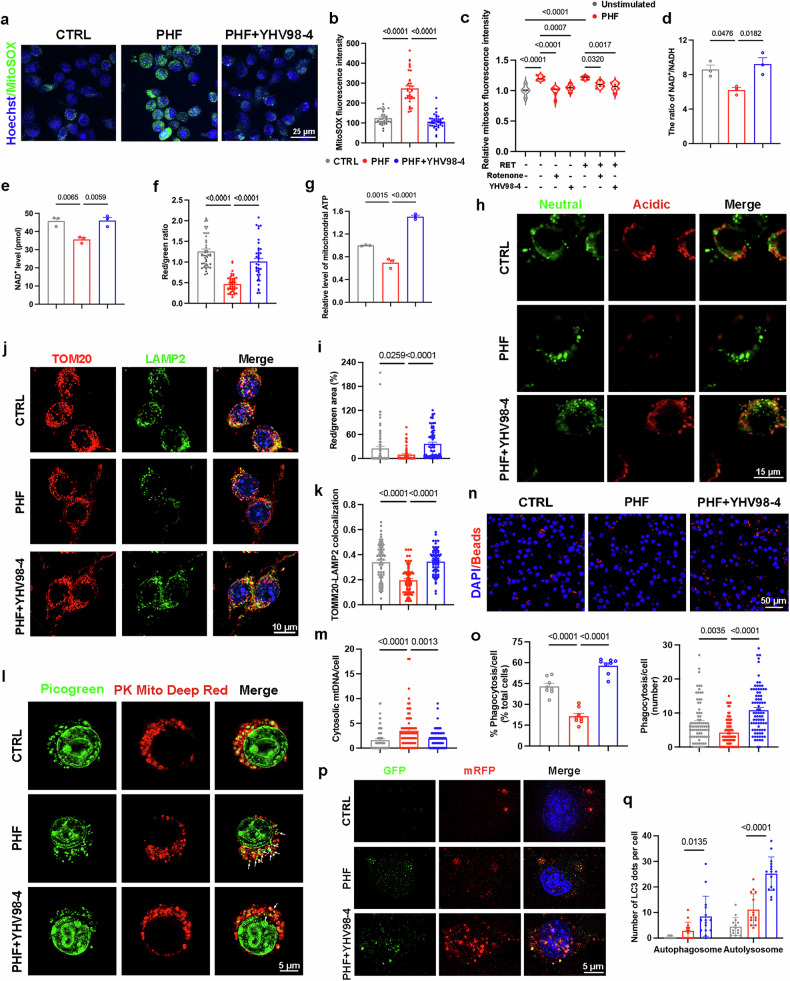
YHV98-4 ameliorates microglial mitochondrial oxidative stress, increases ATP production, and enhances microglial mitophagy, phagocytic and autophagic efficiency. **a**,**b** Representative immunofluorescence images and quantification of mitochondrial ROS levels in BV2 cells treated with PHF or co-incubated with YHV98-4 (*n* = 37–40 cells from three independent experiments). **c** A quantification of MitoSOX fluorescence intensity under different conditions in BV2 cells (*n* = 3 independent experiments). **d**,**e** Measurement of NAD^+^/NADH ratio (**d**) and NAD^+^ level (**e**) in BV2 cells (*n* = 3 independent experiments). **f** A quantification of the cellular fluorescent intensity ratio of red/green (*n* = 40-46 cells from three independent experiments). **g** A quantification of mitochondrial ATP levels in BV2 cells (*n* = 3 independent experiments). **h**,**i** Representative images of mt-mKeima-expressing BV2 cells (**h**) and quantification of the pixel area in the red channel and normalized to the green channel (neutral) (**i**) (*n* = 80 cells from three independent experiments). The red channel represents mitolysosomes, while the green channel represents total mitochondria. **j**,**k** Representative images (**j**) and quantification (**k**) of BV2 cells costained with TOM20 (red) and LAMP2 (green) (*n* = 70 cells from three independent experiments). **l**,**m** Representative STED microscopy images (**l**) and quantification (**m**) of BV2 cells costained with MitoTracker (red) and PicoGreen (green) (*n* = 80 cells from three independent experiments). Picogreen was used to stain dsDNA. PHF was used at 1 μg/ml, and YHV98-4 was used at 20 μΜ for all in vitro experiments. **n**,**o** Representative immunofluorescence images of BV2 cells showing beads uptake (red) (**n**), quantification of the number of beads phagocytized per cell (*n* = 80 cells from three independent experiments), and the proportion of BV2 cells that phagocytosed beads relative to total cells (*n* = 8 fields of view from three independent experiments) (**o**) in PHF-treated conditions with or without YHV98-4. **p**,**q** Representative images of BV2 cells transfected with GFP-mRFP-LC3 plasmids (**p**) and quantification of the number of GFP^+^RFP^+^ autophagosomes and GFP^−^RFP^+^ autolysosomes in BV2 microglia (**q**) (*n* = 14-15 cells from three independent experiments). PHF was used at 1 μg/ml, and YHV98-4 was used at 20 μΜ for all in vitro experiments. The data were calculated using a one-way ANOVA followed by a Tukey’s post hoc analysis and presented as the mean ± s.e.m. The *P* values are presented on the graph.

Having demonstrated the role of Hv1 in microglial ETC dysfunction in tauopathy mice, we next examined whether inhibition of Hv1 could restore mitochondrial function in BV2 microglial cells under tau pathology conditions. To evaluate intracellular ROS levels, we utilized the fluorescent dyes 2′,7′-dichlorodihydrofluorescein diacetate (DCFH-DA) and DHE staining. PHF-stimulated BV2 cells exhibited a significant increase in total ROS and superoxide anion levels, which were markedly reduced following YHV98-4 treatment (Supplementary Fig. 7a–c). Using the MitoSOX probe, we next investigated mitochondrial ROS (mtROS) levels and showed that YHV98-4-treated microglia decreased mtROS production compared with PHF-stimulated microglia (Fig. 6a,b), indicating that Hv1 inhibition ameliorates mitochondrial oxidative stress. RET is another key mechanism contributing to excessive mtROS production^52^. Even without pharmacological induction of RET by the complex V inhibitor oligomycin and the complex II substrate succinate, PHF alone induced RET, as evidenced by the reduction in mtROS following complex I inhibition with rotenone. Notably, YHV98-4 effectively blocked RET (Fig. 6c and Supplementary Fig. 7d). To further assess mitochondrial function, we assessed NAD^+^/NADH levels, which are associated with complex I activity and energy metabolism. Given the established correlation between NAD^+^ levels and AD^53^, we found that YHV98-4 restored NAD^+^ level and normalized the NAD^+^/NADH ratio in PHF-stimulated BV2 cells (Fig. 6d,e). In addition, we evaluated MMP using JC-1 dye, which exists as red aggregates in healthy mitochondria but shifts to green monomers upon MMP loss. YHV98-4 significantly reversed MMP in PHF-treated microglia (Fig. 6f and Supplementary Fig. 7e). Furthermore, we isolated microglial mitochondria and confirmed that YHV98-4 significantly enhanced mitochondrial ATP production after PHF stimulation (Fig. 6g).

Emerging studies highlight the crucial role of mitophagy in maintaining mitochondrial homeostasis and its therapeutic potential in AD^54–56^. To further investigate mitophagy, we utilized the pH-sensitive fluorescent reporter mt-mKeima. Mitophagy flux was quantified as the ratio of red fluorescence area (acidic mitolysosomes) to green fluorescence area (neutral mitochondria). YHV98-4 rescued the impaired mitophagy flux in PHF-stimulated microglia (Fig. 6h, i). In addition, costaining for the mitochondrial marker TOM20 and lysosomal marker LAMP2 revealed reduced colocalization in PHF-stimulated BV2 cells, which was significantly restored after YHV98-4 treatment (Fig. 6j,k). Impaired mitophagy is known to trigger mitochondrial DNA (mtDNA) leakage, consequently causing neuroinflammation^8,9,33,57,58^. We assessed cytosolic mtDNA release and observed a marked increase in PHF-stimulated BV2 cells, which was significantly reduced upon YHV98-4 treatment (Fig. 6l,m).

Both phagocytosis and autophagy are energy-intensive processes essential for tau clearance, relying on mitochondrial function^4,43,59^. Using amine-modified polystyrene latex beads, we evaluated BV2 microglial phagocytosis capacity and found that YHV98-4 effectively rescued the phagocytosis deficits observed in tau pathology (Fig. 6n, o). To validate autophagic flux, we transfected BV2 cells with GFP–mRFP-tagged LC3 plasmid. This construct features pH-sensitive GFP (quenched in acidic lysosomes), pH-resistant mRFP, and the autophagy marker LC3. The yellow puncta (GFP^+^RFP^+^) represent autophagosomes, which transition to red autolysosome upon fusion with lysosomes. PHF induced autophagic defects in microglia, whereas YHV98-4 promoted autophagosome formation and sustained unimpeded autophagic flux (Fig. 6p, q). In summary, these findings indicated that YHV98-4 mitigates microglial mitochondrial oxidative stress, restores energy production, and rescues deficits in phagocytosis, mitophagy and autophagy.

### YHV98-4-treated microglia mitigate tau-induced neuronal damage via intercellular microglia-to-neuron mitochondrial transfer

**Fig. 7:**
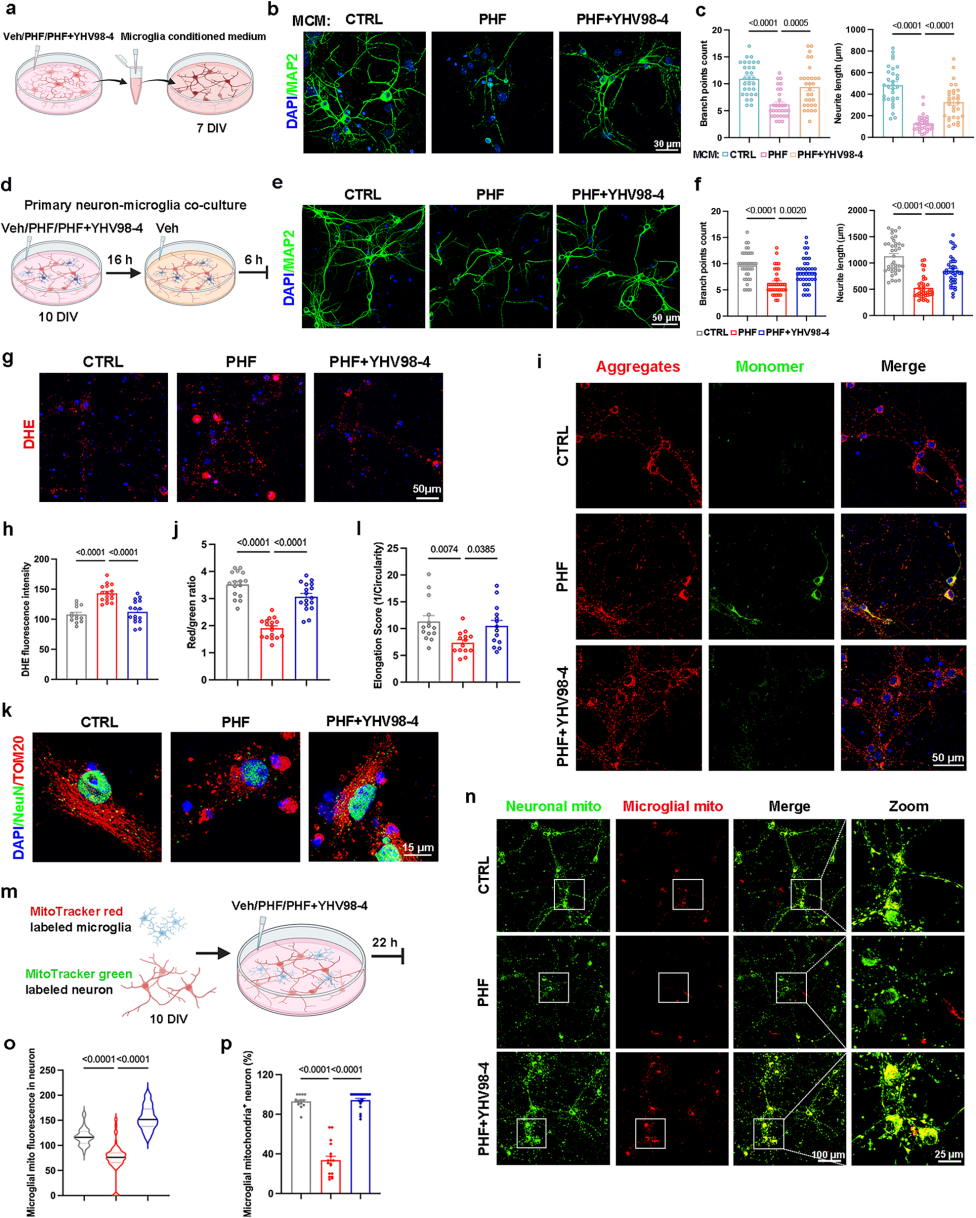
YHV98-4-treated microglia prevent tau-induced neuronal damage through enhanced functional mitochondrial donation to neurons. **a** A schematic diagram illustrating the experimental procedure of neuron treatment with microglia-conditioned medium. **b** Representative immunofluorescence images of neurons incubated with naive, PHF- with or without YHV98-4-treated microglia-conditioned medium and stained with MAP2. **c** A quantification of the number of branch points and total neurite length per cell across different conditions (*n* = 30 cells from three independent experiments). **d** A schematic diagram showing the experimental procedure of neurons coculture with microglia. **e** Representative immunofluorescence images of naive, PHF- with or without YHV98-4-treated neurons and microglia stained with MAP2. **f** A quantification of the number of branch points and total neurite length per cell across different conditions (*n* = 37–39 cells from three independent experiments). **g** Representative immunofluorescence images of naive neurons and microglia, PHF with or without YHV98-4-treated neurons and microglia, stained with DHE. **h** A quantification of the fluorescent intensity of DHE (*n* = 13–16 fields of view from three independent experiments). **i** Representative immunofluorescence staining of JC-1 in primary neurons cocultured with microglia under naive, PHF- or PHF + YHV98-4-treated conditions. **j** A quantification of the ratio of red/green fluorescence intensity in neuronal mitochondria (*n* = 16–18 fields of view from three independent experiments). **k** Representative images of primary neurons coculture with microglia under naive, PHF- or PHF + YHV98-4-treated conditions, costained with TOM20 (red) and NeuN (green). **l** A quantification of mitochondrial elongation in primary neurons (*n* = 14 fields of view from three independent experiments). **m** A schematic diagram showing the experimental procedure of mitochondrial transfer. **n** Representative immunofluorescence images showing microglial mitochondria (red) transferred into neuronal soma and axons, where they fused with neuronal mitochondria (green) under naive, PHF- or PHF + YHV98-4-treated conditions. **o** A quantification of the transferred microglial MitoTracker fluorescence intensity in neurons (*n* = 94–103 cells from three independent experiments). **p** A quantification of the percentage of neurons containing transferred microglial mitochondria (*n* = 16-20 fields of view from three independent experiments). The data were calculated using a one-way ANOVA followed by a Tukey’s post hoc analysis and presented as the mean ± s.e.m. The *P* values are presented on the graph.

Finally, we sought to investigate the impact of Hv1 inhibition on microglial regulation of neuronal health. We demonstrated that healthy microglia alleviated PHF-induced neuronal damage, as evidenced by increased neurite branching and length, as well as reduced ROS levels (Supplementary Fig. 8a–e). We then stimulated neurons with microglial-conditioned medium and revealed that YHV98-4 treatment prevented PHF-stimulated microglia from producing neurotoxic conditioned media that induce neuronal damage (Fig. 7a–c). Moreover, coculturing neurons with microglia revealed that YHV98-4 effectively prevented neurons from PHF-induced damage and reduced oxidative stress (Fig. 7d–h). Significantly, direct treatment of neurons with YHV98-4 alone did not confer any protective effects against PHF-induced neuronal damage (Supplementary Fig. 8f–h), indicating that the neuroprotective effect of YHV98-4 depends on its action on microglia rather than a direct effect on neurons.

Recent studies suggest that mitochondria can be transferred between microglia and neurons. Given our previous findings that tau pathology induces mitochondrial dysfunction and that YHV98-4 ameliorates these deficits, we next investigated whether YHV98-4 rescued mitochondrial function in microglia and promoted the transfer of healthy mitochondria to damaged neurons, thereby mitigating neuronal injury. Interestingly, coculturing PHF-treated neurons with healthy microglia protects neuronal mitochondrial integrity, as evidenced by increased MMP and restored mitochondrial structure (Supplementary Fig. 8i–l). Similarly, YHV98-4 treatment increased neuronal MMP and recovered mitochondrial morphology (Fig. 7i–l), reflecting that YHV98-4 effectively restores mitochondrial health in microglia, thereby contributing to the repair of PHF-induced mitochondrial damage in neurons. To directly assess mitochondrial transfer, we labeled microglial mitochondria and examined their transfer to neurons. The results showed that PHF reduced mitochondrial transfer, whereas YHV98-4 significantly promoted microglia-to-neuron mitochondrial transfer (Supplementary Fig. 9a–d). Furthermore, we separately labeled neuronal and microglial mitochondria before coculturing and observed that YHV98-4 treatment enhanced the transfer of microglia-derived mitochondria and their fusion with neuronal mitochondria (Fig. 7m–p and Supplementary Fig. 9e). In addition, live-cell real-time imaging revealed mitochondrial transfer from neurons to microglia after YHV98-4 treatment (Supplementary Fig. 9e). Together, our data suggested that YHV98-4 restored microglial mitochondrial health, enhanced mitochondrial transfer to neurons and promoted fusion with neuronal mitochondria, thereby mitigating neuronal mitochondrial damage and protecting neurons from tau-induced damage.

## Discussion

Hv1 is a voltage-gated proton channel highly selective for H^+^. Its discovery in microglia has expanded research avenues in the CNS^16^. Hv1 has been implicated in multiple CNS disorders, including spinal cord injury, brain injury, ischemic stroke, multiple sclerosis and Parkinson’s disease^14,17,18^. However, despite the pivotal role of oxidative stress in AD, the specific function of Hv1 and its potential as a therapeutic target remain unexplored. In this study, we found that Hv1 is upregulated in both tauopathy mice and 3×Tg AD mice, accompanied by microglial activation, whereas Hvcn1 deletion or Hv1 inhibition restored microglial homeostasis. Microglial RNA-seq data suggest that TLR-NF-κB and/or the TLR-IRF-IFN-I signaling pathways may contribute to Hv1 upregulation. Specifically, RNA-seq revealed increased activation of pathways such as ‘activation of IRF3/IRF7 mediated by TBK1/IKKε’, ‘IKK complex recruitment’ and proinflammatory cytokine production in AAV-hTAU mice, which were reversed by Hv1 inhibition (Fig. 3f and Supplementary Fig. 4g). Previous studies have shown that tau can trigger cGAS–STING and IFN-I responses via cytosolic mtDNA leakage^9^, consistent with our finding that Hv1 inhibition reduces mtDNA leakage in PHF-stimulated microglia. In addition, Hv1 has been identified as a key regulator of IFN-I production in plasmacytoid dendritic cells^60^, and tau-induced NF-κB activation has been implicated in driving both neuroinflammation and tau spreading^4^, consistent with Hv1’s role in AD pathology. In addition, excessive ROS generation and inflammatory cytokine release, often triggered by mitochondrial dysfunction, can damage ETC and lead to proton leakage and intracellular proton accumulation^61,62^. This acidic environment may further induce Hv1 overexpression, perpetuating a vicious cycle of Hv1-mediated ROS production, inflammation and mitochondrial damage. These events could ultimately form a self-amplifying pathological loop^14^. Further studies will be required to delineate the precise transcriptional mechanisms by which these pathways regulate *Hvcn1* expression in microglia under tauopathy.

Hv1 plays a crucial role in microglial ROS generation by facilitating H^+^ extrusion to sustain NOX2 activity, thereby promoting continuous ROS production^14,16,18^. Although mitochondria are another major source of ROS, especially under AD pathology, the precise mechanisms linking Hv1 to mitochondrial dysfunction remain unclear. Increasing evidence suggests that mitochondrial ROS production in microglia is significantly elevated in AD, leading to mitochondrial damage and impaired bioenergetics^5,63^. However, the mechanistic basis of this dysfunction and potential therapeutic interventions remain poorly understood. Our microglial RNA-seq data revealed extensive ETC disruption in AD, characterized by decreased complex activity, impaired electron transport and diminished ATP synthesis (Fig. 5a, b and Supplementary Fig. 6). Notably, Hv1 inhibition significantly rescued these abnormalities, restoring complex I and complex V function and reestablishing MMP (Fig. 5c, d and Supplementary Fig. 6). Given that complex I dysfunction exacerbates ROS production and oxidative stress, these findings suggest that Hv1 influences the electrochemical gradient and ETC efficiency, ultimately impacting mitochondrial function.

While studies on Hv1-mitochondria interactions remain limited, Patel et al.^64^ revealed that Hv1 regulates mitochondrial ROS production by modulating complex I activity in renal medullary thick ascending limb cells. Similarly, Coe et al.^65^ discovered that Hv1 is expressed in CD8^+^ T cell mitochondria, where it modulated proton flux, oxidative phosphorylation and ATP generation. However, whether and how Hv1 exerts similar effects in microglial mitochondria remains unclear. Given the central role of mitochondrial complex I in ROS generation, its dysfunction in AD results in electron leakage, excessive ROS production and oxidative damage to mitochondria^50,66^. In addition, complexes I, III and IV act as proton pumps, transferring protons from the mitochondrial matrix to the inner membrane’s cytoplasmic side, thereby generating an electrochemical essential for ATP synthesis and normal mitochondrial function^5,67^. Our findings indicate that Hv1 inhibition restored complex I activity, reduces ROS production and rescues complex V function, thereby enhancing ATP synthesis (Fig. 5i,j and Fig. 6). Furthermore, it reestablishes MMP, alleviates mitophagy defects, decreases mtDNA release and enhances ATP-dependent processes such as phagocytosis and autophagy (Fig. 6). Taken together, these results identify Hv1 as a critical regulator of ETC function, mitochondrial integrity and bioenergetic homeostasis in AD. Targeting Hv1 could provide a novel therapeutic avenue to restore mitochondrial function and mitigate AD-associated immune-metabolic dysfunction.

Our study demonstrates that Hv1 plays a critical role in tau hyperphosphorylation and spreading, and its inhibition alleviates tau pathology. While neuronal tau pathology has been extensively studied, emerging evidence suggests that microglia actively participate in tau spreading and clearance through phagocytosis and the autophagy–lysosomal pathway^4,59^. We found that both *Hvcn1*^*−/−*^ and YHV98-4 treatment significantly decrease p-tau levels (Figs. 2a–d and 3a,b and Supplementary Fig. 4a,b). Microglial mitochondria dysfunction under tau pathology leads to impaired ATP production, thereby compromising energy-dependent tau clearance mechanisms such as phagocytosis and autophagy. Following YHV98-4 treatment, mitochondrial functions were restored, leading to enhanced microglial phagocytosis and autophagy efficiency, which could partially explain the reduction in p-tau in the hippocampus (Fig. 6n–q). Moreover, proinflammatory cytokines such as IL1 and IL6, which are known to promote tau phosphorylation and toxicity, were elevated in microglia under tau pathology but were significantly downregulated upon YHV98-4 treatment^4,59^ (Fig. 3f and Supplementary Fig. 4g). These findings highlight Hv1 as a key regulator of tau pathology and a potential therapeutic target.

The discovery of both innate and adaptive immune cells in the brain marks a significant milestone. While microglia are the predominant immune cells in the CNS, other immune cells are also present at low levels, establishing a potential communication network with the parenchyma^68,69^. Microglia function as antigen-presenting cells, actively interacting with adaptive immune cells to regulate immune surveillance and maintain homeostasis^46^. Although previous studies have linked Hv1 to microglia-mediated neuroinflammation through promoting cytokine production and metabolic reprogramming^17,70^, its role in coordinating innate and adaptive immune response remained unclear. Our findings reveal that Hv1 activation drives microglial transformation into a proinflammatory state (MHC II^+^), leading to excessive T cell infiltration and activation, thereby creating a neurotoxic immune environment (Figs. 2i–l and 3j–m and Supplementary Figs. 2a,b and 3b,c). Importantly, we identified Hv1 as a key regulator linking innate and adaptive immunity and demonstrated that its inhibition by YHV98-4 effectively disrupts pathological microglia–T cell crosstalk, attenuates neuroinflammation, and promotes neuroprotection (Fig. 3k–m and Supplementary Fig. 4n–q).

Our microglial transcriptomic data further revealed that Hv1 plays a pivotal role in synaptic integrity, as extensive synaptic damage observed in AD mice was markedly reversed following YHV98-4 treatment (Figs. 3g,h and 4l,m and Supplementary Fig. 4h,i). Recent research has revealed that microglia establish direct interactions with neurons via TNTs^13^. TNTs are transient membrane channels that facilitate the transfer of organelles, plasma membrane components, nucleic acids, cytokines, signaling molecules and pathological protein aggregates between cells and serve as primary conduits for mitochondrial transfer^71,72^. Healthy microglia transfer functional mitochondria to neurons burdened with α-synuclein or toxic tau aggregates through TNTs, thereby promoting neuronal recovery. By contrast, only a modest amount of mitochondria is transferred from neurons to microglia through TNTs^13^. Notably, when microglial mitochondrial function is impaired, such as by oligomycin A-induced complex III dysfunction, neuronal rescue fails^13^. In our study, tau pathology impaired the transfer of mitochondria from microglia to neurons. By contrast, inhibition of microglial Hv1 rescues microglial mitochondrial function and significantly enhanced mitochondrial transfer to neurons, thereby rescuing neuronal mitochondria l integrity and mitigating neuronal injury (Fig. 7 and Supplementary Figs. 8i–l and 9). Live-cell real-time imaging also revealed mitochondrial transfer from neurons to microglia following YHV98-4 treatment, which might be attributed to the clearance of damaged neuronal mitochondrial by microglia under PHF stimulation. Although TNTs, gap junctions and extracellular vesicles have all been implicated in mitochondrial transfer, the precise mechanism by which YHV98-4 enhances this process under tau pathology remains to be elucidated. These findings suggest that targeting Hv1 may provide a novel strategy for AD intervention by restoring microglial mitochondrial function and facilitating functional mitochondria transfer to neurons.

Despite these insights, our study has several limitations. First, while we confirmed Hv1 localization in mitochondria and demonstrated its impact on the ETC function, further validation using mitochondrial electrophysiology is required and is currently underway. Second, consistent with previous results, we confirmed that Hv1 is predominantly expressed in microglia rather than neurons or astrocytes in the CNS. To further investigate its role in AD, we are generating microglia-specific *Hvcn1*^*−/−*^ mice (*Hvcn1*^*fl/fl*^:*Cx3cr1*^*CreER2*^) and crossing them with AD models for targeted investigations. Finally, as Hv1 is also expressed in T cells, it is imperative to clarify its role in brain-infiltrated T cells to fully understand its impact on neuroinflammation and neurodegeneration.

## Supplementary information

Supplementary Information

## Data Availability

Data from this study are available upon a reasonable request to the corresponding author.
