# Supplementary material for: Hv1 inhibition rescues AD pathology by restoring microglial mitochondrial function and enhancing mitochondrial transfer
Source: Exp Mol Med. 2025 Dec 17;57(12):2833–51. doi: 10.1038/s12276-025-01593-z (PMC12800102; doi:10.1038/s12276-025-01593-z)
Supplement: Supplementary file 1 — Supplementary Information [file 12276_2025_1593_MOESM1_ESM.pdf]

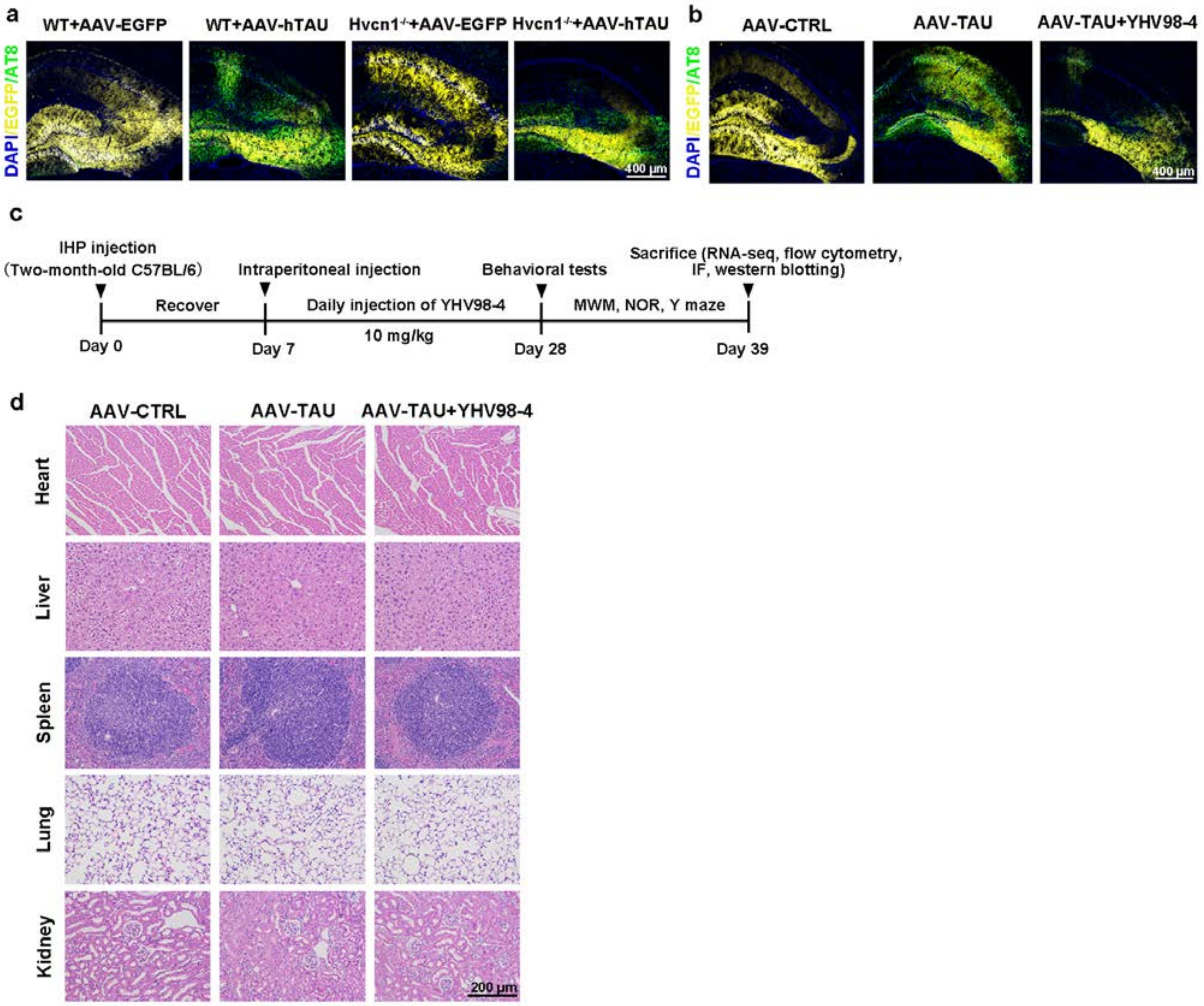

Supplementary Fig. 1 Characterization of intrahippocampal injection and YHV98-4 treatment without inducing histopathological changes

a, Representative image of AAV-EGFP (yellow) and AT8 (green) expression and distribution in the hippocampus of WT+AAV-EGFP, WT+AAV-hTAU, *Hvcn1*$^{-/-}$+AAV-EGFP, and *Hvcn1*$^{-/-}$+AAV-hTAU mice. **b,** Representative image of AAV-EGFP (yellow) and AT8 (green) expression and distribution in the hippocampus of AAV-CTRL, AAV-TAU, and AAV-TAU+YHV98-4 mice. **c,** Schematic overview of the intrahippocampal injection and YHV98-4 treatment protocol. **d,** No observable morphological abnormalities in the heart, liver, spleen, lungs, or kidneys following 21 days of intraperitoneal YHV98-4 administration in AAV-TAU mice.

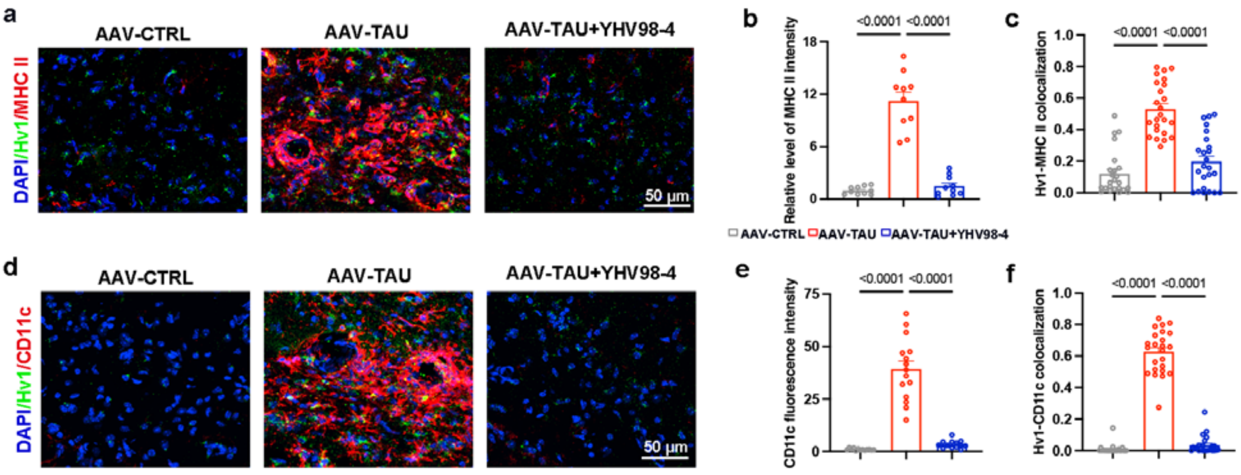

Supplementary Fig. 2 Inhibition of Hv1 reduces CD11c$^+$ and MHC II$^+$ microglial subsets

a, b, Representative immunofluorescence images of Hv1 (green) co-stained with MHC II (red) and DAPI (blue) (a) and quantification of MHC II intensity (b) in the hippocampus of AAV-CTRL, AAV-TAU, and AAV-TAU+YHV98-4 mice (n = 10 fields of view from 3 mice). **c,** Quantification of the colocalization of MHC II with Hv1 in the hippocampus of AAV-CTRL, AAV-TAU, and AAV-TAU+YHV98-4 mice (n = 24 cells from 3 mice). **d, e,** Representative immunofluorescence images showing Hv1 (green) co-stained with CD11c (red) and DAPI (blue) (d) and quantification of CD11c intensity (e) (n = 10 fields of view from 3 mice). **f,** Quantification of the colocalization of CD11c with Hv1 in the hippocampus of AAV-CTRL, AAV-TAU, and AAV-TAU+YHV98-4 mice (n = 24 cells from 3 mice). Data were calculated using one-way ANOVA followed by Tukey’s post hoc test and are presented as mean ± SEM. *P* values are presented on the graph.

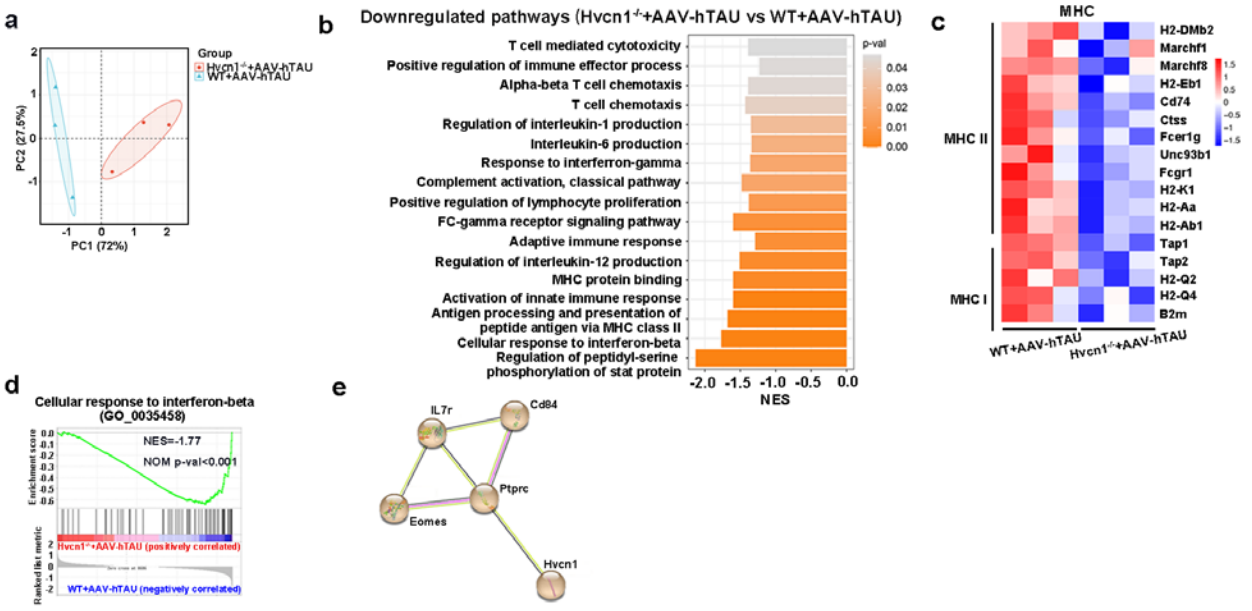

Supplementary Fig. 3 ***Hvcn1*** **deletion reduces neuroinflammation in tauopathy mice.**

a, Principal component analysis showing separation of RNA-seq data between WT+AAV-hTAU and *Hvcn1*$^{-/-}$+AAV-hTAU groups. **b,** GSEA analysis of gene sets downregulated in *Hvcn1*$^{-/-}$+AAV-hTAU compared to WT+AAV-hTAU groups. **c,** Heatmap of MHC-related gene expression in *Hvcn1*$^{-/-}$+AAV-hTAU versus WT+AAV-hTAU groups. **d,** GSEA plot showing enrichment of "Cellular response to interferon-beta" in WT+AAV-hTAU compared to *Hvcn1*$^{-/-}$+AAV-hTAU groups. **e,** Protein interaction network of Hv1 and immune response genes downregulated in *Hvcn1*$^{-/-}$+AAV-hTAU versus WT+AAV-hTAU.

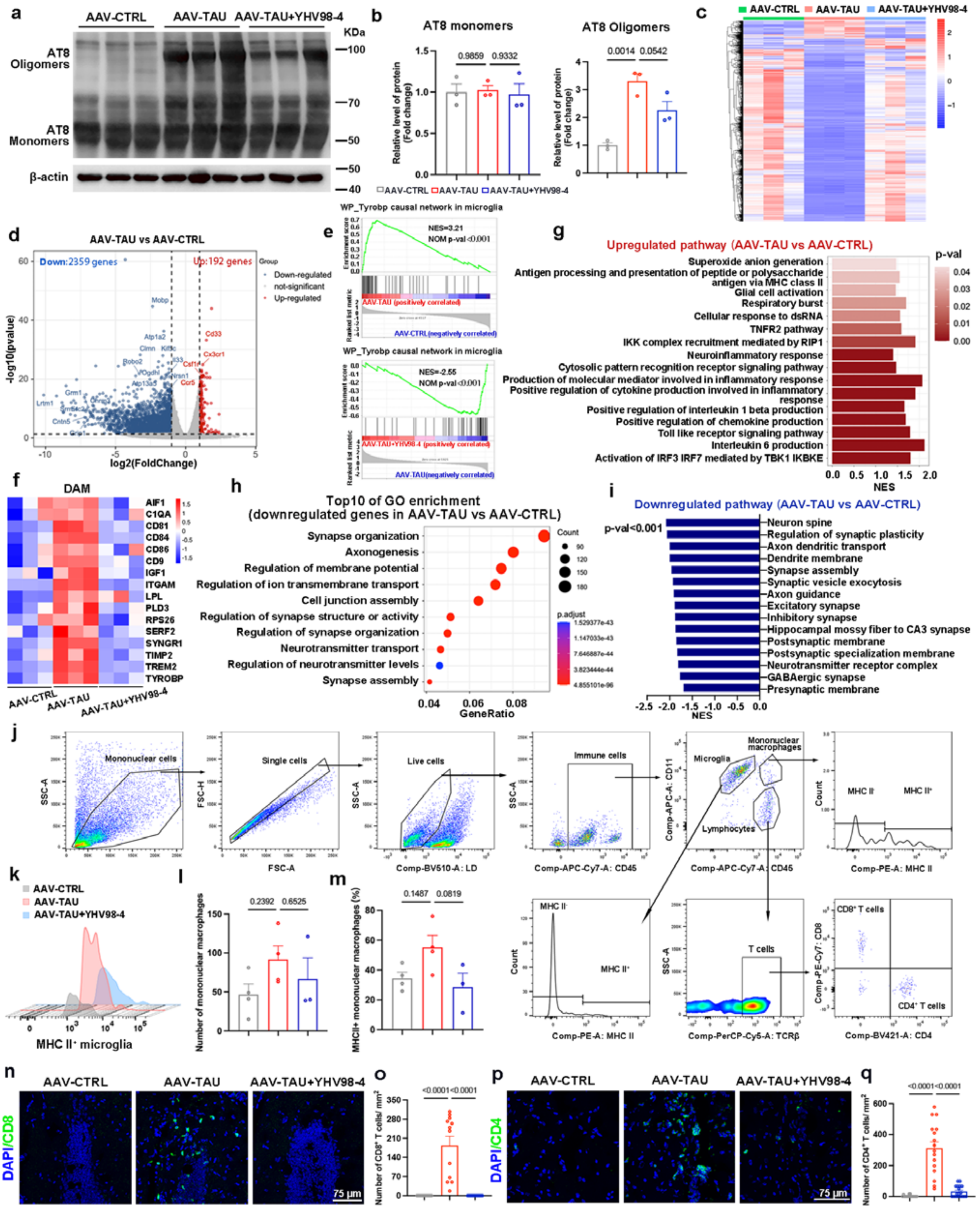

Supplementary Fig. 4 YHV98-4 reduces AT8 oligomer levels and T-cell infiltration in AAV-TAU mice.

a, Western blot showing AT8 oligomers and monomers in hippocampal lysates from AAV-CTRL, AAV-

TAU, and AAV-TAU+YHV98-4 mice. **b,** Quantification of AT8 monomer and oligomer levels in the hippocampus (*n* = 3). **c,** Heatmap of DEGs across AAV-CTRL, AAV-TAU, and AAV-TAU+YHV98-4 groups. **d,** Volcano plots of DEGs from AAV-TAU versus AAV-CTRL groups (*n* = 3). Selected genes were labeled. **e,** GSEA plots depicting enrichment of "Tyrobp causal network in microglia" in AAV-TAU mice compared to AAV-CTRL mice and AAV-TAU+YHV98-4 mice compared to AAV-TAU mice. **f,** Heatmap of DEGs related to DAM in AAV-CTRL, AAV-TAU, and AAV-TAU+YHV98-4 groups. **g,** GSEA of major inflammation-related pathways upregulated in AAV-TAU versus AAV-CTRL. **h,** Top 10 GO enrichment terms of downregulated DEGs in AAV-TAU versus AAV-CTRL. **i,** GSEA of the significantly downregulated pathways enriched in microglia of AAV-TAU versus AAV-CTRL mice (p-val<0.001). **j,** Flow cytometry gating strategy for the analysis of AAV-CTRL, AAV-TAU, and AAV-TAU+YHV98-4 mice. **k,** Flow cytometry analysis of MHC II^+ microglia. **l, m,** Quantification of mononuclear macrophage numbers in the hippocampus and cortex identified by $CD45^{hi}$ $CD11b^{hi}$ cells (l) and the proportion of MHC II^+ mononuclear macrophages among hippocampus and cortex mononuclear macrophages (m). The number of live cells has already been downsampled to the same level when stained with Zombie Aqua before comparing mononuclear macrophage numbers among AAV-CTRL (*n* = 4), AAV-TAU (*n* = 3), and AAV-TAU+YHV98-4 (*n* = 3) mice. **n-q,** Representative immunofluorescence staining of $CD8^+$ (n) and $CD4^+$ (p) T cells (green) in the hippocampus, and quantification of their respective cell densities (o for $CD8^+$: 12 fields of view from 3 mice; q for $CD4^+$: 16–18 fields of view from 3 mice). Data were calculated using one-way ANOVA followed by Tukey’s post hoc analysis and are presented as mean ± SEM. *P* values are presented on the graph.

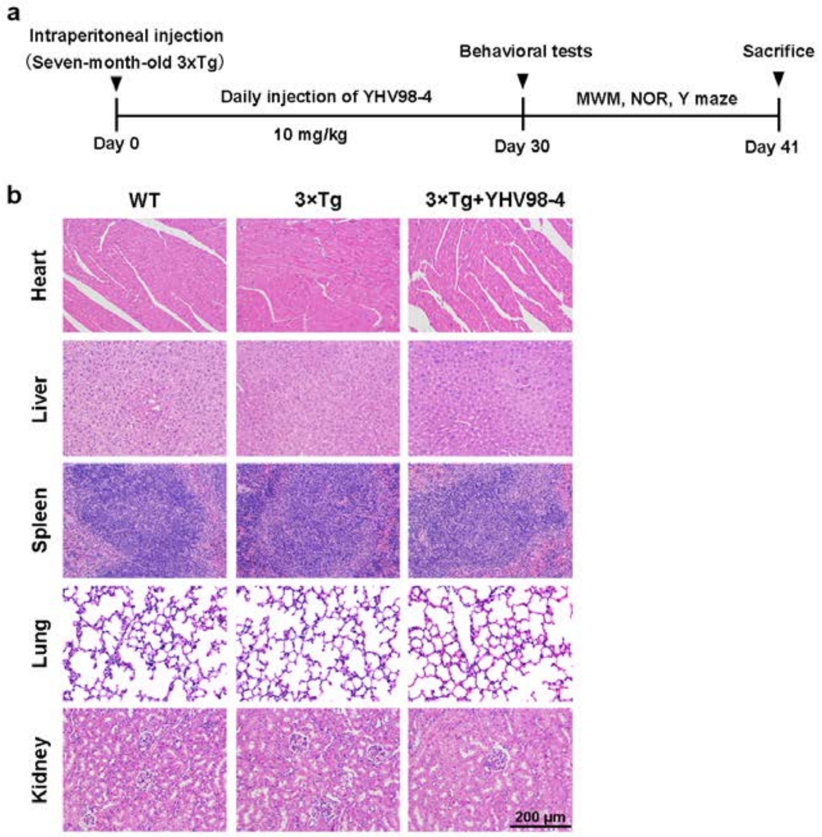

Supplementary Fig. 5 YHV98-4 can be safely delivered to 3×Tg AD mice.

a, Schematic overview of the YHV98-4 treatment in 3×Tg mice. **b,** No observable morphological abnormalities were detected in the heart, liver, spleen, lungs, or kidneys following 1 month of intraperitoneal YHV98-4 administration in 7-month-old 3×Tg mice.

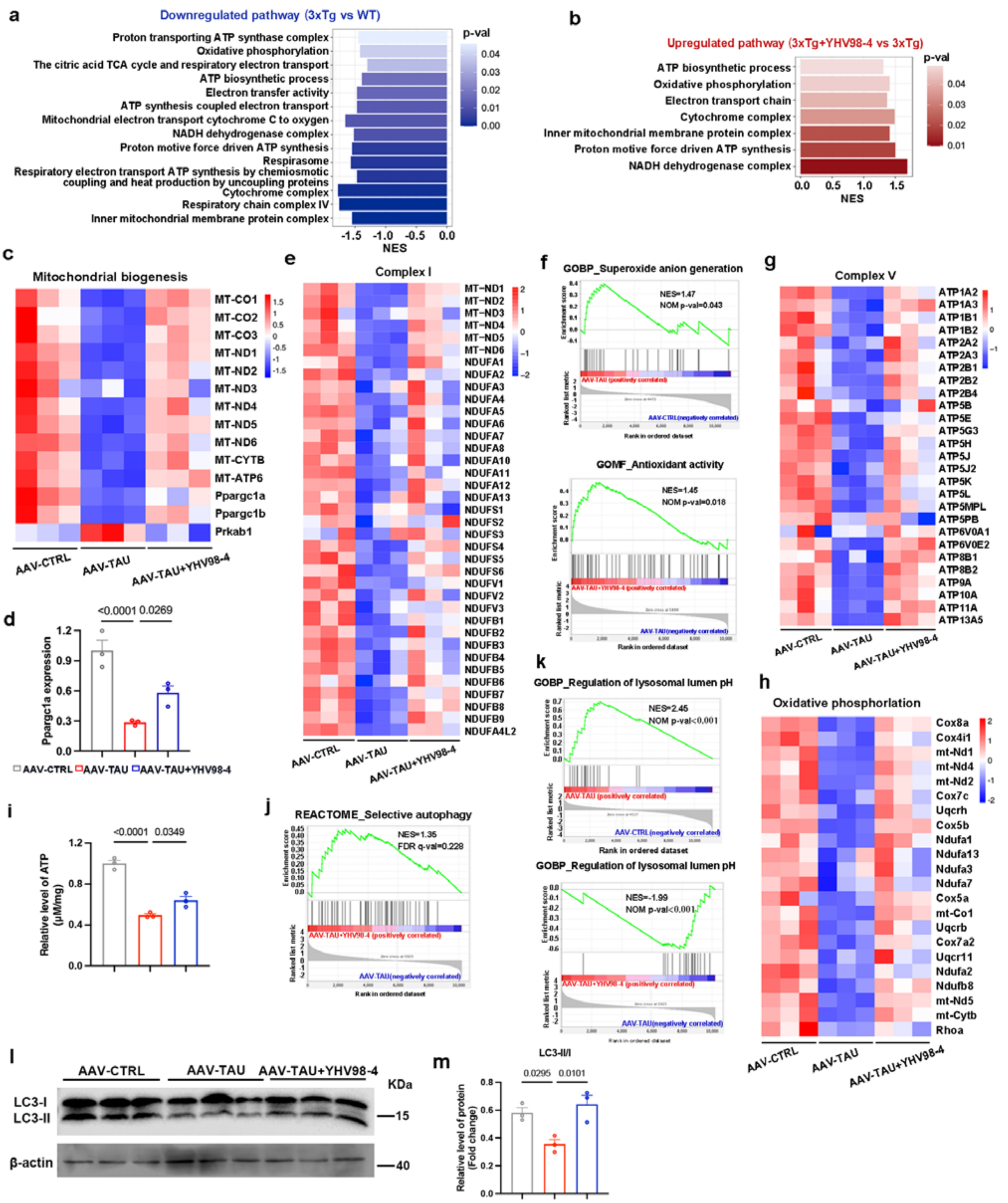

Supplementary Fig. 6 ETC is disrupted in AD mice and restored by YHV98-4 treatment.

a, GSEA showing significantly downregulated mitochondria-associated pathways in 3×Tg mice compared to WT mice **b,** GSEA showing significantly upregulated mitochondria-associated pathways in 3×Tg+YHV98-4 mice compared to 3×Tg. **c, d,** Heatmap of DEGs related to mitochondrial

biogenesis (c) and RNA-seq analysis of mRNA expression levels of Ppargc1a (d) in AAV-CTRL, AAV-TAU, and AAV-TAU+YHV98-4 groups. **e,** Heatmap of DEGs related to Complex I in AAV-CTRL, AAV-TAU, and AAV-TAU+YHV98-4 groups. **f,** GSEA plots showing enriched "Superoxide anion generation" in AAV-TAU vs. AAV-CTRL, and "Antioxidant activity" in AAV-TAU+YHV98-4 vs. AAV-TAU. **g, h,** Heatmap of DEGs related to Complex V (g) and oxidative phosphorylation (h) in AAV-CTRL, AAV-TAU, and AAV-TAU+YHV98-4 groups. **i,** Quantification of ATP levels normalized to protein concentration in AAV-CTRL, AAV-TAU, and AAV-TAU+YHV98-4 mice (*n* = 3 per group). **j,** GSEA plots showing upregulation of "Selective autophagy" in AAV-TAU+YHV98-4 compared to AAV-TAU mice. **k,** GSEA plots showing that "Regulation of lysosomal lumen pH" was upregulated in AAV-TAU compared to AAV-CTRL, but downregulated in AAV-TAU+YHV98-4 compared to AAV-TAU mice. **l, m,** Western blot of LC3-I and LC3-II in hippocampal lysates (l) and quantification of the LC3-II/LC3-I ratio (m) from AAV-CTRL, AAV-TAU, and AAV-TAU+YHV98-4 mice (*n* = 3). Data were calculated using one-way ANOVA followed by Tukey's post hoc analysis and are presented as mean ± SEM. *P* values are presented on the graph.

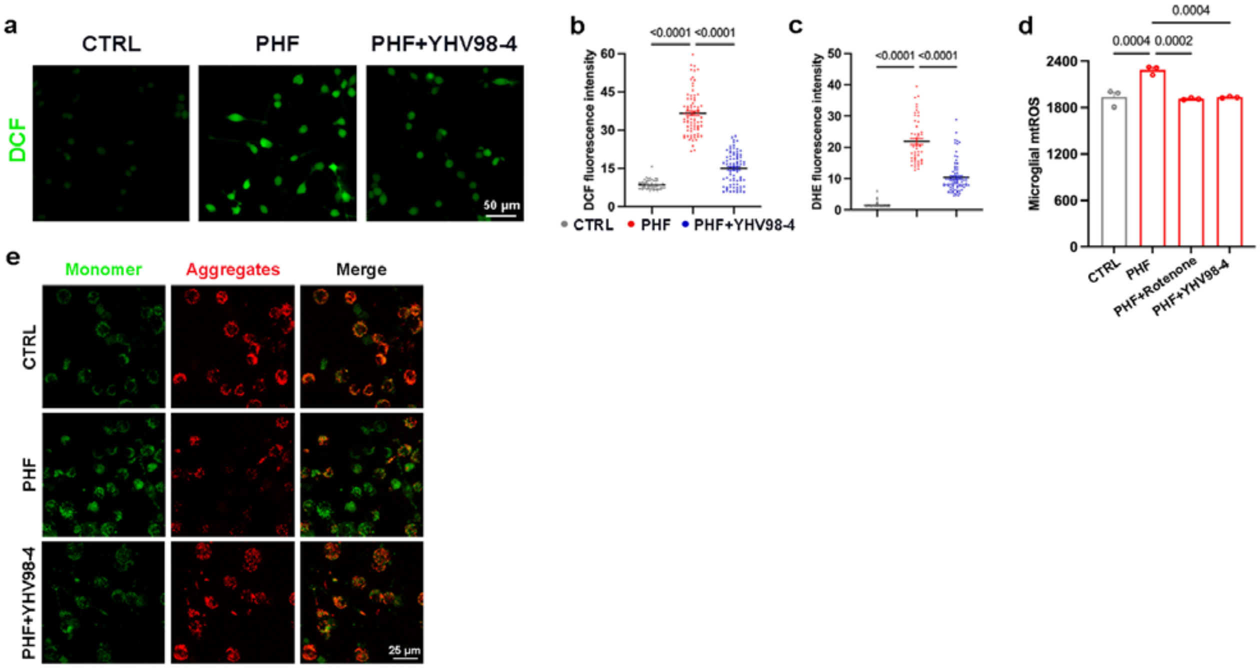

Supplementary Fig. 7 Inhibiting Hv1 restores mitochondrial function.

a-c, Representative immunofluorescence images of BV2 cells showing ROS levels detected by DCFH-DA (a), quantification of cellular DCF fluorescence intensity (b, *n* = 60-80 cells from 3 independent experiments); and quantification of DHE fluorescence intensity (c, *n* = 50-80 cells from 3 independent

experiments). **d,** Flow cytometry analysis of mtROS levels in BV2 cells under different conditions (*n* = 3). **e,** Representative immunofluorescence images of JC-1 in BV2 cells. PHF (1 μg/mL) and YHV98-4 (20 μM) were used for all *in vitro* experiments. Data were calculated using one-way ANOVA followed by Tukey's post hoc analysis and are presented as mean ± SEM. *P* values are presented on the graph.

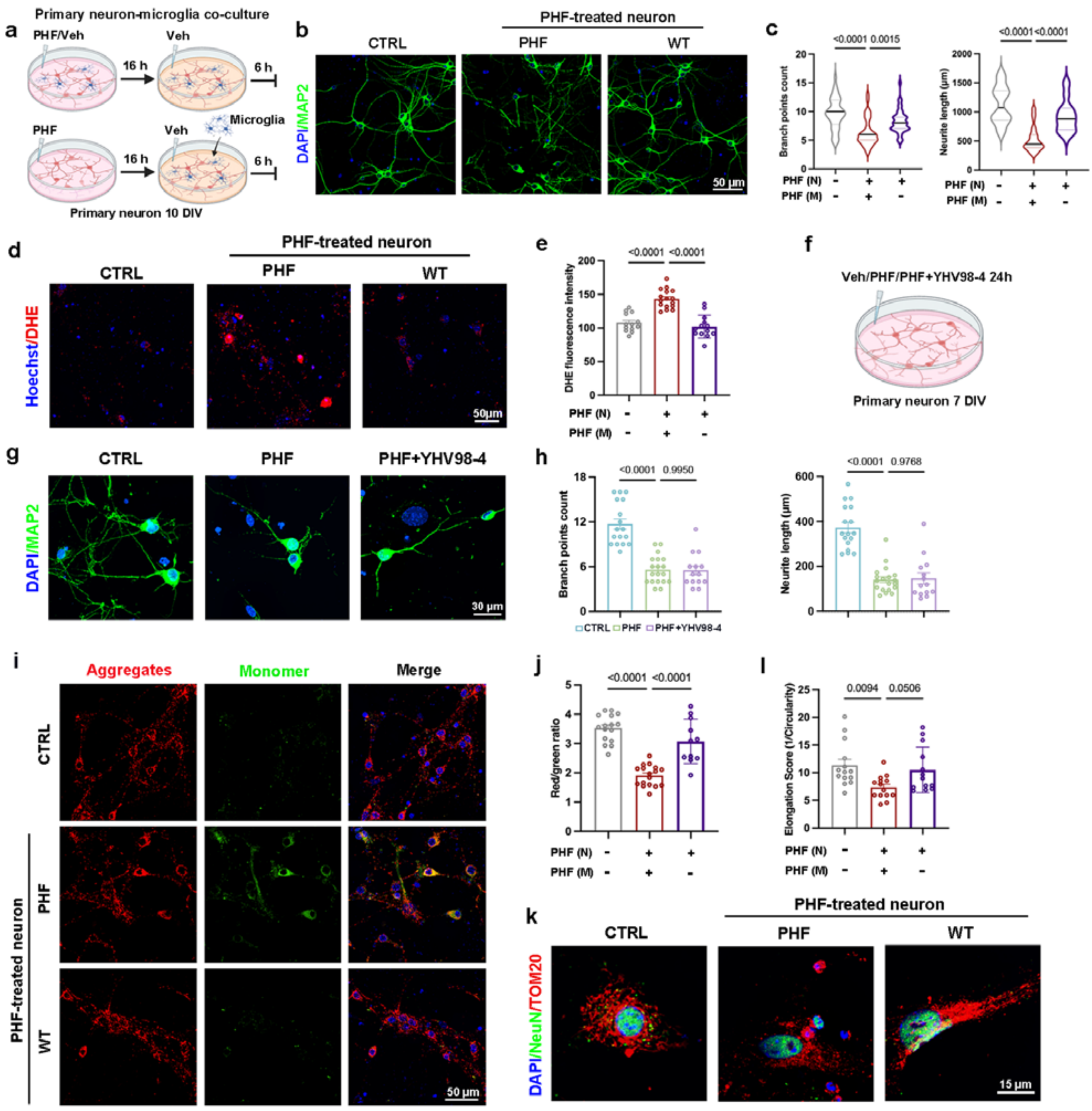

Supplementary Fig. 8 Healthy microglia ameliorate PHF-induced neuronal mitochondrial damage and rescue neurons.

a, Schematic diagram illustrating the experimental procedure of neuron co-culture with microglia. **b**,

Representative immunofluorescence images of naive neurons and microglia, PHF-treated neurons and microglia, and PHF-treated neurons co-cultured with naive microglia, all stained with MAP2. **c**, Quantification of the number of branch points and total neurite length per cell across different conditions (*n* = 37-40 cells from 3 independent experiments). **d,** Representative immunofluorescence images of naive neurons and microglia, PHF-treated neurons and microglia, and PHF-treated neurons co-cultured with naive microglia, all stained with DHE. **e**, Quantification of the fluorescent intensity of DHE (*n* = 13-16 fields of view from 3 independent experiments). **f,** Schematic diagram showing the experimental procedure of direct stimulation of primary neurons. **g,** Representative immunofluorescence images of naive, PHF, PHF+YHV98-4-treated neurons, stained with MAP2. **h,** Quantification of the number of branch points and total neurite length per cell across different conditions (*n* = 14-19 cells from 3 independent experiments). **i**, Representative immunofluorescence staining of JC-1 in naive neurons and microglia, PHF-treated neurons and microglia, and PHF-treated neurons co-cultured with naive microglia. **j**, Quantification of the red/green fluorescence intensity ratio of neuronal mitochondria (*n* = 11-18 fields of view from 3 independent experiments). **k**, Representative images of naive neurons and microglia, PHF-treated neurons and microglia, and PHF-treated neurons co-cultured with naive microglia, co-stained with TOM20 (red) and NeuN (green). **l**, Quantification of mitochondrial elongation in primary neurons (*n* = 13-14 fields of view from 3 independent experiments). Data were calculated using one-way ANOVA followed by Tukey's post hoc analysis and presented as mean ± SEM. *P* values are presented on the graph.

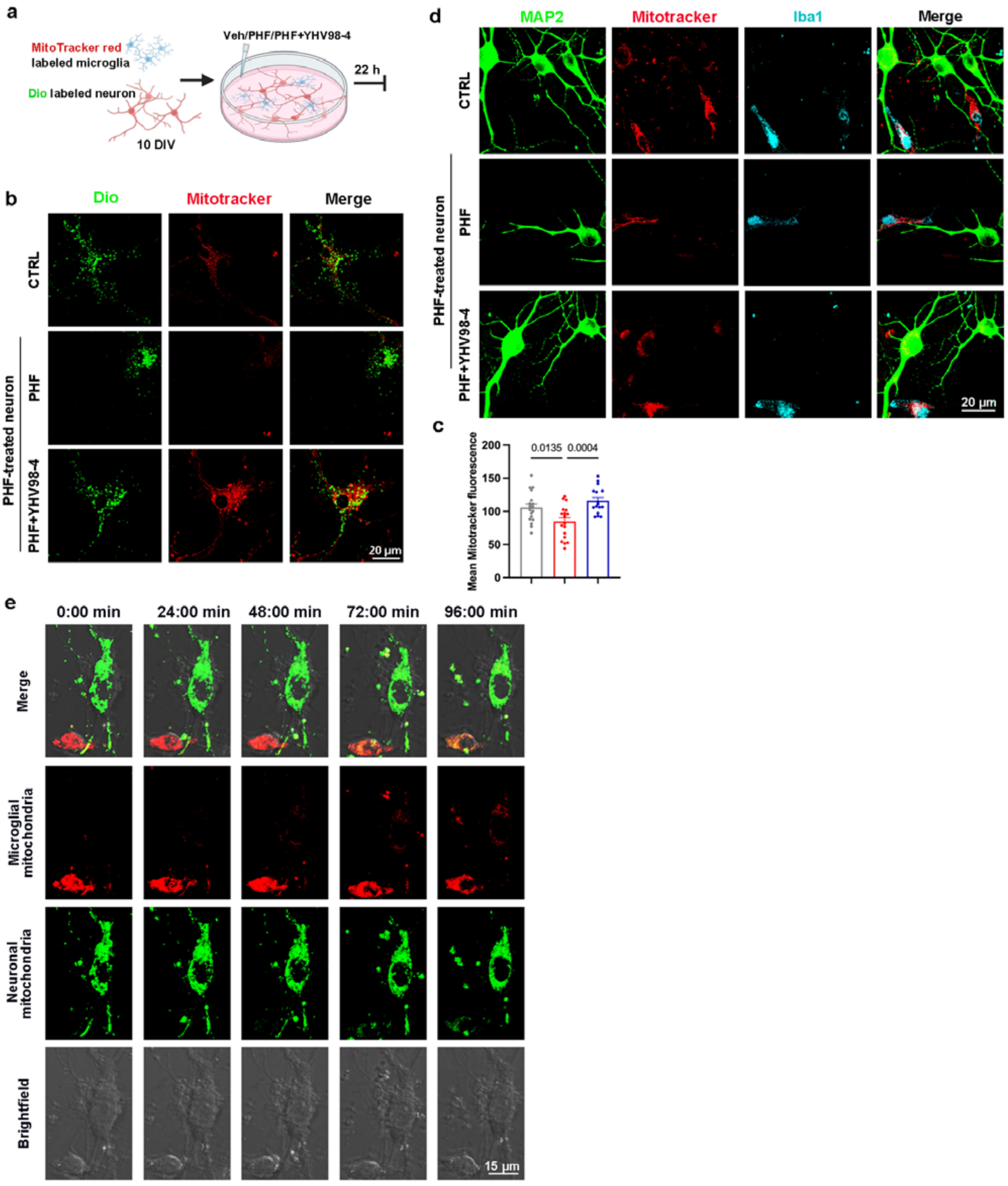

Supplementary Fig. 9 YHV98-4 enhances microglial mitochondrial transfer to neurons following PHF stimulation

a, Schematic diagram showing the experimental procedure of mitochondrial transfer. **b**, Representative

immunofluorescence images showing microglial mitochondria (red) transferred into Dio-labeled neurons (green) under naive, PHF with or without YHV98-4-treatment. **c**, Quantification of the transferred microglial MitoTracker fluorescence intensity in neurons (*n* = 16-19 cells from 3 independent experiments). **d,** Representative images showing the transfer of microglial mitochondria to neurons under naive, PHF, or PHF+YHV98-4-treated conditions. Microglia were labeled with Iba1, microglial mitochondria with Mitotracker, and neurons with MAP2. **e,** Live-cell imaging showing mitochondrial transfer between microglia and neurons following PHF+YHV98-4 treatment. Data were calculated using one-way ANOVA followed by Tukey's post hoc analysis and presented as mean ± SEM. *P* values are presented on the graph.
